# BatteryDataExtractor: battery-aware text-mining software embedded with BERT models†

**DOI:** 10.1039/d2sc04322j

**Published:** 2022-09-23

**Authors:** Shu Huang, Jacqueline M. Cole

**Affiliations:** Cavendish Laboratory, Department of Physics, University of Cambridge J. J. Thomson Avenue Cambridge CB3 0HE UK jmc61@cam.ac.uk; ISIS Neutron and Muon Source, Rutherford Appleton Laboratory, Harwell Science and Innovation Campus Didcot Oxfordshire OX11 0QX UK

## Abstract

Due to the massive growth of scientific publications, literature mining is becoming increasingly popular for researchers to thoroughly explore scientific text and extract such data to create new databases or augment existing databases. Efforts in literature-mining software design and implementation have improved text-mining productivity, but most of the toolkits that mine text are based on traditional machine-learning-algorithms which hinder the performance of downstream text-mining tasks. Natural-language processing (NLP) and text-mining technologies have seen a rapid development since the release of transformer models, such as bidirectional encoder representations from transformers (BERT). Upgrading rule-based or machine-learning-based literature-mining toolkits by embedding transformer models into the software is therefore likely to improve their text-mining performance. To this end, we release a Python-based literature-mining toolkit for the field of battery materials, BatteryDataExtractor, which involves the embedding of BatteryBERT models in its automated data-extraction pipeline. This pipeline employs BERT models for token-classification tasks, such as abbreviation detection, part-of-speech tagging, and chemical-named-entity recognition, as well as new double-turn question-answering data-extraction models for auto-generating repositories of inter-related material and property data as well as general information. We demonstrate that BatteryDataExtractor exhibits state-of-the-art performance on the evaluation data sets for both token classification and automated data extraction. To aid the use of BatteryDataExtractor, its code is provided as open-source software, with associated documentation to serve as a user guide.

## Abstract 2

BatteryDataExtractor is the first property-specific text-mining tool for auto-generating databases of materials and their property, device, and associated characteristics. The software has been constructed by embedding the BatteryBERT model.

## Graphical abstract

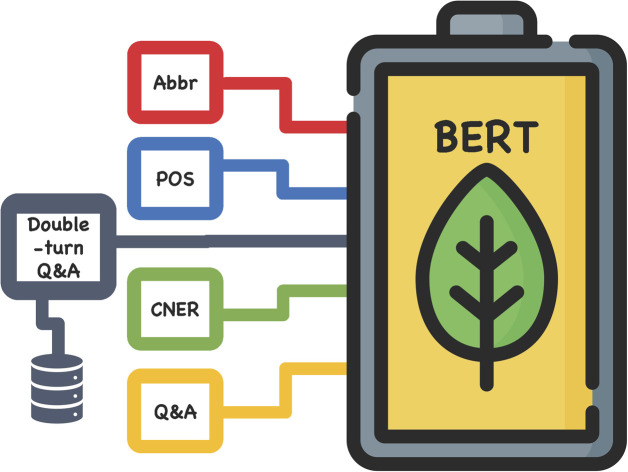

## 1 Introduction

Scientific publications have long been a critical source of information for researchers to gain insights into the latest findings of scientific endeavor and use them to accelerate data-driven discoveries. In the area of materials science, for example, successful data-driven techniques have been applied to the design of new materials such as catalysts,^1,2^ solar cells,^3–5^ nuclear materials,^6,7^ and battery materials.^8–11^ Key to these materials discoveries is the quality and quantity of data. While computationally generated databases have led to the spin-off of many materials-discovery projects since the launch of the Materials Genome Initiative,^12–15^ literature-based data extraction is becoming increasingly popular to make use of the latest literature data to create new databases or to augment existing materials databases.^16,17^

Compared to other data sources, literature-text data are fully processed (as supposed to raw data) and readily accessible in electronic format, while their total number perpetually increases with advancing time. However, scientific-literature texts are lengthy, diverse and unstructured, which makes it difficult for researchers to screen the literature in order to obtain useful information. Literature mining is thus becoming of high demand for scientific-information retrieval and knowledge extraction. Efforts have thus been invested into text mining by manually labeling hundreds of scientific papers^11,18,19^ to serve supervised or semi-supervised machine-learning (ML) methods that automate large-scale database curation such as synthetic parameters;^20–22^ while natural-language-processing (NLP) methods and ML methods have been employed to auto-generate materials properties.^23–27^ Apart from data extraction, text mining can also assist in the reviewing of research trends^28–30^ and provide latent scientific information using unsupervised ML methods.^31–33^

In order to improve the efficiency and effectiveness of literature mining and adapt it to a specific materials domain such as batteries, several studies have been dedicated to the development of the chemistry-aware toolkit, *e.g.* ChemDataExtractor^34,35^ and PDFDataExtractor,^36^ whose functionalities are based on NLP and ML algorithms. For example, ChemDataExtractor v1.3 embraces a hybrid system for chemical-named-entity recognition (CNER), including regular expression-based, dictionary-based, and conditional-random-field (CRF)-based^37^ recognizers. It also uses rule-based phrase parsing and table parsing to enable database auto-generation.^34^

Recent years have witnessed a particularly rapid development of text mining and NLP technologies^38^ due to the introduction of huge deep-learning models, such as long short-term memory (LSTM)^39^ and bidirectional-encoder representations from transformers (BERT).^40^ Transformer-based language models have achieved state-of-the-art results on almost all downstream NLP tasks, such as named-entity recognition and question-answering.^40,41^ Huge transformer models have also been created in the area of scientific literature by training on subject-specific data. Models such as MatBERT^42^ and MatSciBERT^43^ have demonstrated their usage on tasks including text classification and CNER. Meanwhile, the BatteryBERT^44^ language model has provided domain-specific capabilities within materials science; thereby, it can classify papers into those that concern battery materials or otherwise, as well as distinguish the type of battery material that has been mentioned as belonging to an anode, cathode or electrolyte. The aforementioned ‘chemistry-aware’ toolkit, ChemDataExtractor, can also be updated by embedding its capabilities into a transformer model. One example is the latest version of ChemDataExtractor (v2.1), which takes advantage of the fine-tuned SciBERT^45^ language model to achieve both organic and inorganic CNER simultaneously.^46^

However, current efforts that apply transformer models to chemistry-based text-mining processes have remained largely unexplored, partly due to a lack of integrated software. A single toolkit that is designed for automatically extracting text about chemicals and properties from scientific documents that is based on transformer models, is still needed to enhance the productivity of mining scientific text. To this end, we designed a transformer-based data-extraction pipeline by embedding pretrained BatteryBERT models into a Python toolkit, BatteryDataExtractor. To the best of our knowledge, BatteryDataExtractor is the first software that uses a full deep-learning-based pipeline of a language model for the automatic extraction of cognate chemical and property data.

The main contributions of this work are as follows. We release the transformer-based battery-specific literature-mining toolkit, BatteryDataExtractor, whose software architecture is based on that of ChemDataExtractor, but the core part of the architecture has been changed into BERT-based models.^34,35^ The rule-based and ML-based NLP plugins within ChemDataExtractor were replaced by a fine-tuned BatteryBERT models,^44^ including abbreviation detection, part-of-speech (POS) tagging, and CNER, all of which are open source and available online. We also designed a novel double-turn question-answering system to automate the extraction of both materials and property data as well as general information from scientific documents. The BatteryBERT-based automated data-extraction pipeline does not need any manually encoded parsing rules; instead, the tool can be implemented by just a single line of code in BatteryDataExtractor. Both the NLP token-classification plugins and the double-turn question-answering-based data-extraction method achieved better performance than ChemDataExtractor when tested on evaluation data sets. In addition, several functions have been introduced in the BatteryDataExtractor toolkit in order to improve its software user-friendliness, including updated web scrapers, document readers and tokenizers, a database auto-saving option, an original text-saving option, and a device-selection option. Full documentation of the code is also provided to serve as a user guide.

## 2 Implementation details

### 2.1 System overview

**Fig. 1:**
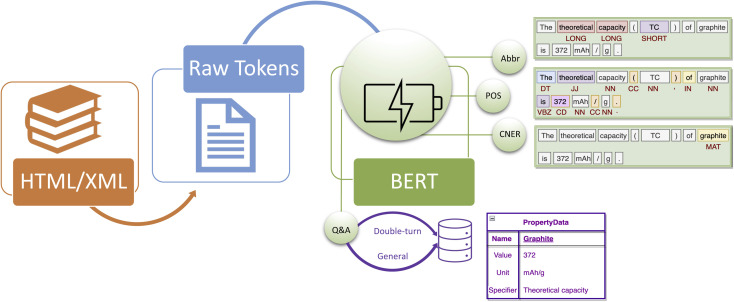
System overview of BatteryDataExtractor. The natural-language-processing pipeline firstly converts the HTML/XML raw text into tokens, which are then fed into BERT models for the downstream tasks, including abbreviation detection, part-of-speech tagging, chemical-named-entity recognition, and question answering. Abbreviation detection identifies the abbreviation words (SHORT) and their long form (LONG). Part-of-speech tagging marks up words as corresponding to a particular part of speech (*e.g.* DT: determiner, JJ: adjective, NN: noun, CC: coordinating conjunction) based on its context. Chemical-named-entity recognition detects the chemical name (MAT). The Q&A system retrieves data, such as the materials-property data, using the double-turn or general question-answering strategy. The retrieved data are saved into the final database.

The system overview of BatteryDataExtractor is shown in Fig. 1. Most stages of the data-extraction pipeline are consistent with those of ChemDataExtractor:^34,35^ the HTML/XML file is converted into raw text by a document reader, which is then processed by several NLP tools including abbreviation detection, POS tagging, CNER, and question-answering-based information extraction. The final output is a database consisting of scientific information such as {material, property} data. Note that the NLP tools which were based on manually encoded rules or ML-based algorithms in ChemDataExtractor have been all embedded into transformer models in the new software. In addition, we introduced a “double-turn” question-answering strategy for the automatic data extraction of materials and properties. Overall, the BatteryBERT-based BatteryDataExtractor tool outperforms the latest ChemDataExtractor on the battery-related evaluation data sets.

### 2.2 Token classification models and data sets

**Fig. 2:**
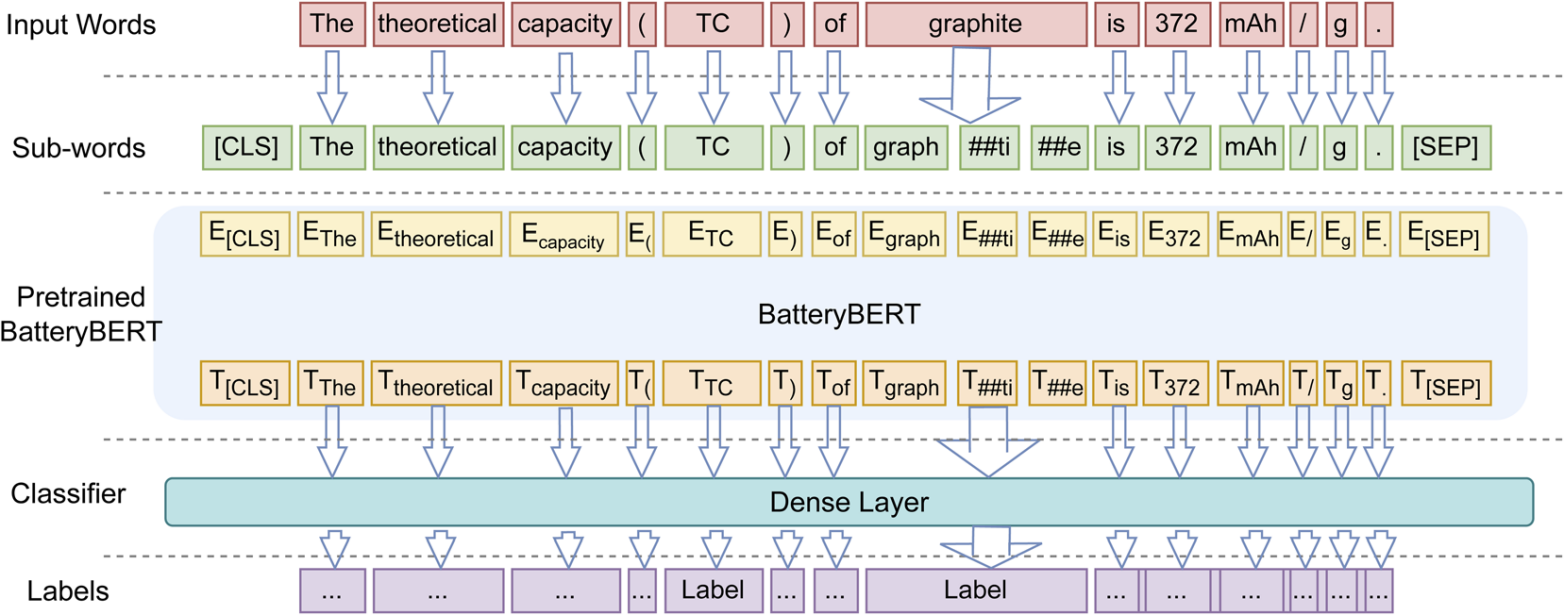
Token-classification-model architecture of BatteryBERT. E represents the input embedding. T represents the contextual representation of token i. [CLS] is the special symbol for classification output, and [SEP] is the special symbol to separate non-consecutive token sequences.

Abbreviation detection, POS tagging and CNER are all essentially token-classification tasks, which can be formulated as a problem where an input sequence of words {w1, w2, w3, w4, …} is processed by the language model to predict as a sequence of output labels {l1, l2, l3, l4, …}. BERT has been demonstrated to produce state-of-the-art performance on text classification by fine-tuning the language model on the specific data sets.^47,48^ In order to extract data within the battery domain, we thus chose the pretrained BatteryBERT model as a starting point for the downstream token-classification tasks. The transfer-learning characteristic of BERT makes it easy to apply BatteryBERT to the token-classification tasks by just adjusting the final layer (dense layer) of the transformer model. Fig. 2 shows the architecture of the fine-tuned token-classification model. The input sentence is firstly tokenized into sub-words which are then fed into BatteryBERT. The WordPiece tokenizer was used for this task; this splits a single word into multiple sub-words according to its frequency of occurrence within the corpus. For example, the word “graphite” in Fig. 2 is split into three sub-words: “graph”, “##it”, and “##e”. Its input embedding is then processed by the pretrained BatteryBERT model into contextual representations, which are, in turn, fed into the final dense layer of the language model to make predictions about the corresponding tokens. The predicted labels vary with different token-classification tasks. In the CNER task, for example, the label of the “graphite” token will be predicted as “MAT”.

The data sets used for training the classifier are shown in Table 1. We adopted the “BIO” tagging scheme to label tokens, where a word/sub-word is labeled as a B-label if it is the beginning of an entity, or an I-label if the word/sub-word is contained inside the same entity. Other tokens are labeled as “O” if they do not belong to any entity. For each task, BatteryBERT was fine-tuned on a mixed data set to generalize its model performance on various kinds of data sets. For example, we trained our models on four different training sets: CHEMDNER, MatScholar, SOFC, and BioNLP.^49–52^ These data sets contain both organic and inorganic materials from different areas of materials science, such as fuel cells and biomaterials. By mixing training data that span various domains, we believe that the fine-tuned CNER module can identify more kinds of chemical names compared to just training on one specific data set.

**Table 1:** Data sets for abbreviation detection, POS tagging, and CNER

Data set type	Data set name	Total number	Ref.
Abbreviation detection	PLOS	1 161 888	54
	SDU@AAAI-21	17 457	55
POS tagging	Conll2003	10 677	56
	The Penn Treebank	3828	57
CNER	CHEMDNER	12 712	49
	MatScholar	5454	50
	SOFC	873	51
	BioNLP	93 515	52

The training hyperparameters and implementation details are as follows. All downstream models were trained with a maximum sequence length of 512 and a batch size of 16 on eight NVIDIA DGX A100 GPUs on the ThetaGPU cluster at the Argonne Leadership Computing Facility (ALCF). We also tested the epoch size from 1 to 15, the batch size {16, 32} and the learning rate {2 × 10^−5^, 3 × 10^−5^, 5 × 10^−5^} for all tasks. The training time was ∼15 minutes for POS tagging, ∼1 h for CNER, and ∼7 h for abbreviation detection.

### 2.3 BatteryBERT-based automated data-extraction model

#### 2.3.1 Double-turn question-answering model

The BatteryBERT-based automated data-extraction model makes use of the fine-tuned BatteryBERT on question-answering data sets, which has also been designed for interactive use at https://www.materialsforbatteries.org/.^44^ This Q&A model was embedded into BatteryDataExtractor in a fashion that we could adopt a “double-turn” question-answering strategy for data extraction. The double-turn question-answering method transforms a relation-extraction task into a problem of detecting answer spans from the context of the text.^53^ For example, we designed a BERT-based material parser that retrieves the answers to two carefully sequenced rounds of questions in order to extract both the material and property data. At the start of this process, users need to specify the property name that one wishes to find. The material parser will then ask the following question based on the provided textual context:

“**What's the value of the ‘PROPERTY_NAME**’?”

Once a valid property value has been found, the second question will be:

“**Which material has a ‘PROPERTY_NAME’ of ‘ANSWER_OF_THE_PREVIOUS_QUESTION’?**”

**Fig. 3:**
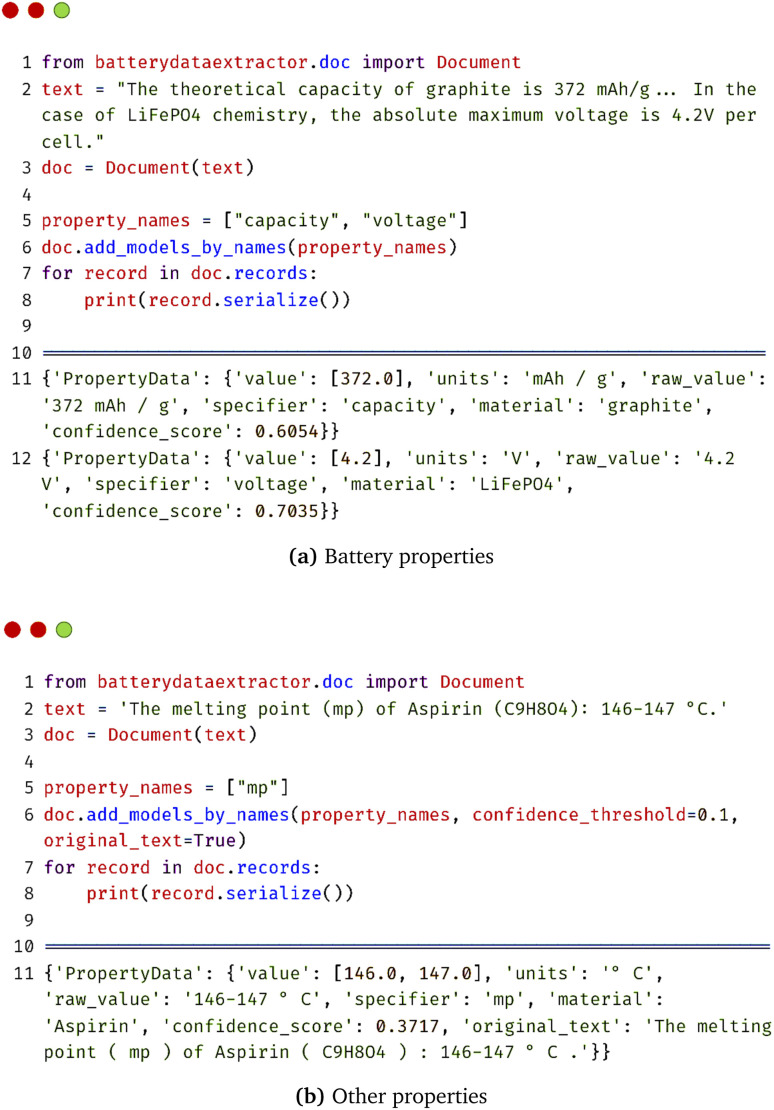
BatteryBERT-based automated data-extraction model for {material, property} data in BatteryDataExtractor.

After that, the relations between the property value and the specific material will be eventually extracted. Fig. 3 shows an example of how the {material, property} data can be extracted with a few lines of code in BatteryDataExtractor. By just providing the property name “capacity” and “voltage” in the “add_models_by_names” function of the Document class (Fig. 3a), data relations can be found as “PropertyData” with several fields including value, unit, raw value, specifier name, and material name. Compared to the previous way of extracting data by manually defining multiple rules in a specific materials domain of interest,^24,34^ this new relation-extraction method greatly reduces the time of human intervention.

In addition, a confidence-score threshold can be set for the double-turn question-answering system, where a higher confidence-score threshold means a higher precision and a lower recall. Hence, it is also much easier to control the data quality and quantity than the rule-based method, in which the model behavior cannot be changed easily once the human-encoded rules have been determined.

Another advantage of the BatteryBERT-based automated model is its model generalizability. As is demonstrated in Fig. 3b, the fine-tuned BatteryBERT can also extract property data in other areas of materials science; for example, the property, “melting point”. This is because the BatteryBERT model is huge and capable of capturing lengthy contextual information, not only about batteries but also about all kinds of materials and their cognate properties, which can similarly be detected and extracted with just a few lines of code. Even though BatteryBERT is not the optimal language model to extract data from another materials domain, since it was not trained on an appropriate domain-specific corpus, Fig. 3b demonstrates the ability and potential of BatteryDataExtractor to extract data about materials and properties other than those associated with battery materials.

#### 2.3.2 General question-answering model

**Fig. 4:**
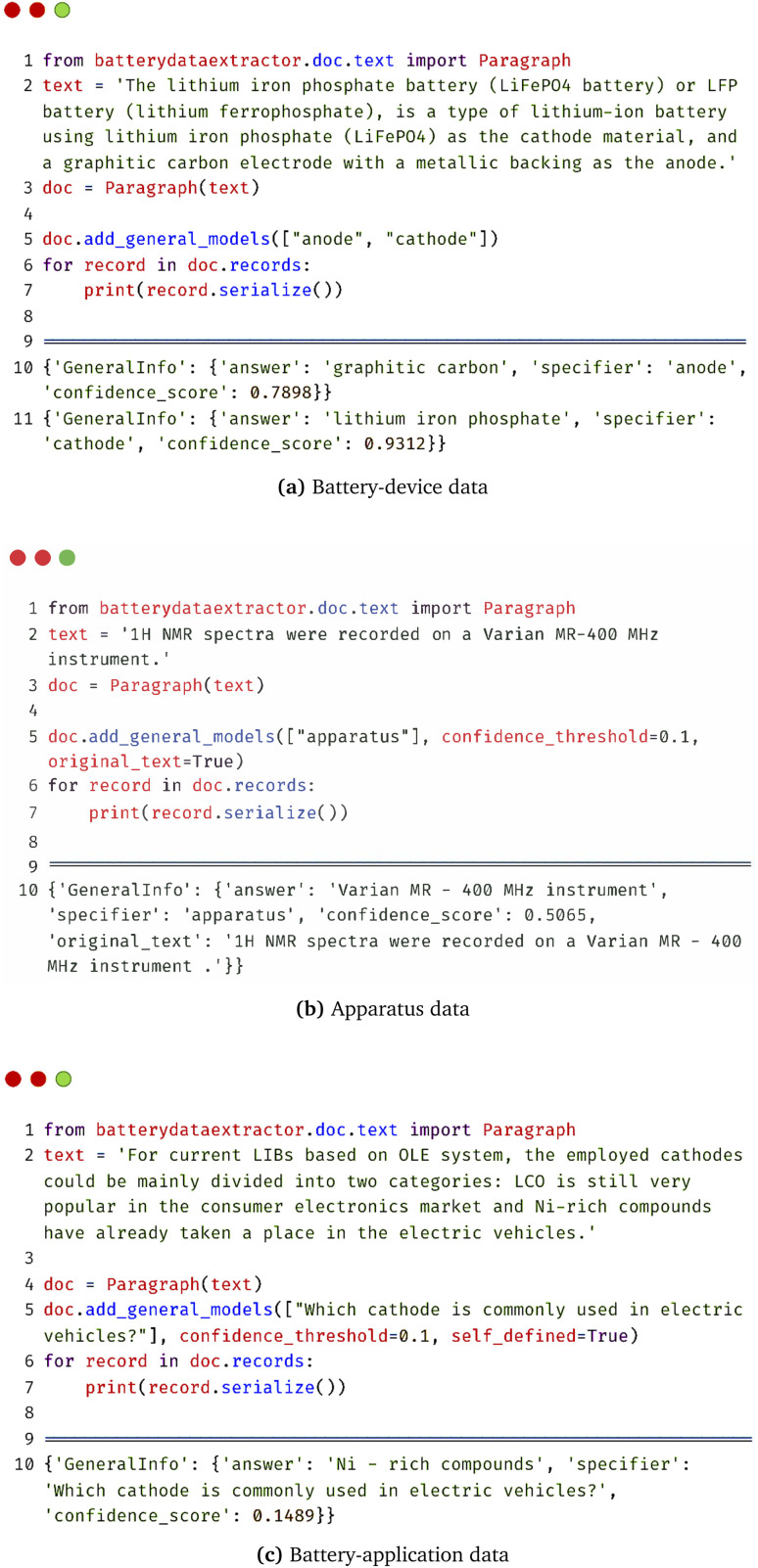
BatteryBERT-based automated data-extraction model for general information in BatteryDataExtractor.

Apart from the extraction of materials and property data, a general parser was also included in BatteryDataExtractor in order to retrieve more general data information. Fig. 4 shows three examples of general information that can be extracted about: battery devices, the application of batteries, and apparatus that have been used in characterizing a material. Fig. 4a exemplifies an instruction for device-based data extraction which has already been demonstrated previously.^44^ Users only need to specify the name or category of the general information in the “add_general_models” Python function, and BatteryDataExtractor can then automatically look for the relevant information that exists in the textual context. It is the same for the non-battery applications (Fig. 4b), in which the name of a materials-characterization apparatus used to define a materials characteristic task is predicted as a final output. Note that our model is able to predict the correct information even when the specifier name is not explicitly present in the textual context (apparatus *versus* instrument). Moreover, instead of inputting only the name of the general information, users can also select a self-defined option to ask any other questions by setting “self_defined” as True. As is shown in Fig. 4c, any question such as: “Which cathode is commonly used in electric vehicles?” can be answered only if the final output has a confidence score higher than that of the threshold. This “add_general_models” function enables BatteryDataExtractor to extract various kinds of data in a complicated setting, which proves its ability to create large and diverse data sets for mining text from the scientific literature.

To summarize, the transformer-based automated data-extraction model is achieved by embedding the fine-tuned question-answering BatteryBERT model into BatteryDataExtractor. A new ‘double-turn’ question-answering strategy was adopted to extract interdependent material and property information. Extracting {material, property} data or general information only requires users to provide the specific name of a property or general information and its corresponding contextual text. In certain situations, users can also obtain the data based on the self-defined questions. This BatteryBERT-based automated model can accelerate the data-extraction process without any requirement to invest in substantial amounts of time and tedium on manually writing rules. The combined use of these material-based and general-information-based tools has huge potential for scientists to conduct various text-mining research. The data-extraction model has also demonstrated decent results on the evaluation data sets, which will be discussed below.

### 2.4 Other NLP features

Several important updates about BatteryDataExtractor are introduced in this section, which are not directly related to the transformer model. Instead, those new NLP features aim to improve the user experience based on the user feedback of ChemDataExtractor over the past few years. Full instructions for users can be found in the code documentation, while a brief overview of those minor updates is given here:

• Web scraper and document reader. The bespoke web scrapers and document readers of ChemDataExtractor have been updated in BatteryDataExtractor according to the latest policies from three publishers (Royal Society of Chemistry, Elsevier, and Springer), including a new file processor for JATS-format XML files.

• Sentence tokenizer. BatteryDataExtractor uses a sentence tokenizer, SciSpacy, which has been specifically trained on scientific text. This tokenization package has been demonstrated to operate robustly on scientific documents.^58^

• Save the database option. The extracted data can be automatically saved into a local database with the text, CSV, and JSON format by just a single line of code. It is not necessary to post-process the data each time before saving to the database.

• Save the original text option. The original text of a document or paper from which the data were extracted can be saved by specifying “original_text = True” when initializing the automated data-extraction model (see, for example, in Fig. 3b and 4b). This update can help to evaluate the accuracy of the database output and check the model performance.

• Choose CPU or GPU. Since BatteryDataExtractor employs an advanced and huge deep-learning model, a high-performance GPU can accelerate its data extraction. Thus, we provide an option for users to specify which device is to be used. The default option remains as CPU, for user convenience.

## 3 Evaluation

### 3.1 Evaluation results for token classification

The common metrics for evaluating a token-classification model are precision, recall and F1-score. Precision represents the proportion of predicted positives that is truly positive. Recall is the proportion of actual positives that is correctly classified. The F1 score combines precision and recall into a single metric. The corresponding equations are given by:1
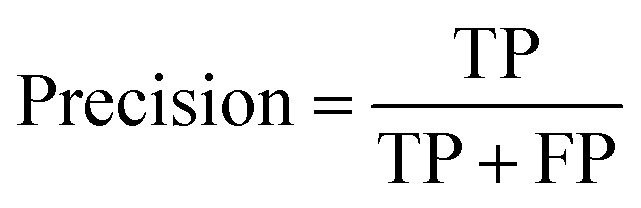
2
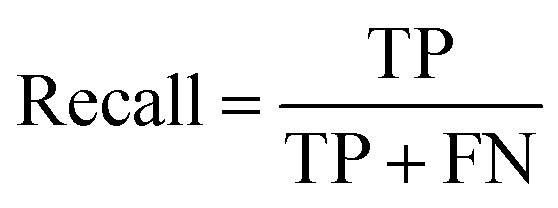
3
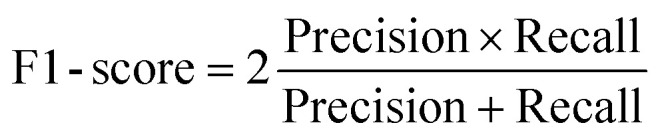
where TP denotes true positive, FP false positive, and FN false negative.

In contrast to other classification tasks, these metrics differ for CNER, POS tagging, and abbreviation detection in that all words need to be predicted correctly in order for a prediction to be counted as correct. We used the Python package, seqeval, for the token-classification evaluation, which is designed for sequence-labeling evaluation that also supports the “BIO” tagging scheme.^59,60^

Table 2 lists the best F1-score of different models on the specific data set. The evaluation details for all hyperparameters can be found in the ESI.† ChemDataExtractor 2.0 was evaluated in order to provide a comparative reference to a rule-based ‘chemistry-aware’ NLP software architecture; version 2.0 is the last version of ChemDataExtractor prior to the introduction of transformer models into its software framework. Nevertheless, the latest ChemDataExtractor version 2.1 was also chosen for evaluation, in which the fine-tuned SciBERT model has been included in the CNER toolkit. The BatteryOnlyBERT-cased models achieved the highest F1 score for abbreviation detection, and the BatteryOnlyBERT-uncased model exhibited the highest F1-score on CNER tasks, while the BERT-base-cased model afforded the best performance on POS tagging. The three best models were embedded into BatteryDataExtractor and have also been released on the Hugging Face website for independent use (https://huggingface.co/batterydata/). For the abbreviation-detection task, the F1 score was not calculated for ChemDataExtractor v2.0/v2.1, since this software can only detect a pair of abbreviation spans when both the short words and their long form exist in a sentence, while BatteryDataExtractor can detect either the abbreviation alone or as a pair. For the CNER model, all of the four BatteryBERT models have a better performance than the original BERT model, which is as expected since they were further pretrained on the battery corpus. The much lower F1 score of ChemDataExtractor v2.0 might be due to the fact that its legacy CNER capabilities were not specifically trained on the data set that was used for fine-tuning the BatteryBERT models. By contrast, the F1-score of ChemDataExtractor v2.1 is slightly lower than that of BatteryDataExtractor, as the former model was also not trained specifically on the fine-tuned CNER datasets. However, ChemDataExtractor v2.1 still performs better than v2.0 on the CNER task due to the nature of deep-learning models. The new training set includes material names that ChemDataExtractor has never seen, such as biomaterials and a range of the inorganic materials. For POS tagging, the reason why the original BERT model demonstrates the best performance relative to other models might be that the POS-tagging training set is not relevant to scientific text; rather, it pertains to a general English-language-based data set. Since the original BERT model was pretrained on a generic English corpus, such as books and Wikipedia text, it is expected to show better evaluation results when tested on a generic English data set. The F1 score was for POS tagging on all the BERT-related models are higher than that of ChemDataExtractor v2.0/v2.1, for the same reason as the evaluation results for the CNER task.

**Table 2:** F1-score of abbreviation detection, CNER, and POS tagging for six BERT-based models, including BatteryBERT, BatteryOnlyBERT, base BERT, as well as that for ChemDataExtractor v2.0 and v2.1

Model	Abbreviation detection	CNER	POS tagging
BatteryBERT-cased	0.9502	0.9584	0.9667
BatteryBERT-uncased	0.9475	0.9578	0.9632
BatteryOnlyBERT-cased	**0.9516**	0.9589	0.9640
BatteryOnlyBERT-uncased	0.9492	**0.9598**	0.9605
BERT-base-cased	0.9491	0.9458	**0.9669**
BERT-base-uncased	0.9464	0.9517	0.9633
ChemDataExtractor v2.0	—	0.6998	0.8649
ChemDataExtractor v2.1	—	0.8882	0.8649

Overall, the BERT and BatteryBERT models outperform ChemDataExtractor v2.0 and v2.1 which encodes the rule-based and SciBERT-based algorithms. Furthermore, we believe that the BatteryBERT-based BatteryDataExtractor can be more reliable for ‘chemistry’ text-mining tasks such as information retrieval and data extraction, especially in the battery domain.

### 3.2 Evaluation results for the BatteryBERT-based automated data-extraction model

The precision and recall metrics were also adopted for the evaluation of the automated data-extraction model. For this framework, we focus on the evaluation of materials-property data extraction based on the double-turn question-answering model. Thereby, precision is the fraction of the correct (“True”) data in the evaluation data set, and recall is the fraction of the data relation that is extracted from the data set (*vide supra* for details).

An evaluation set of materials-property data was sampled from the manually labeled database^24^ consisting of a total of 100 data records of materials with five battery-material properties: capacity, voltage, Coulombic efficiency, energy, and conductivity. Each data record includes the correct material name while its properties all carry their correct corresponding value and units; the original context wherefrom the data are extracted is also provided. We used an “add_models_by_names” function with the property name as the input for BatteryDataExtractor to extract the data according to the contextual text. The property data were then retrieved with confidence scores assigned to them.

**Fig. 5:**
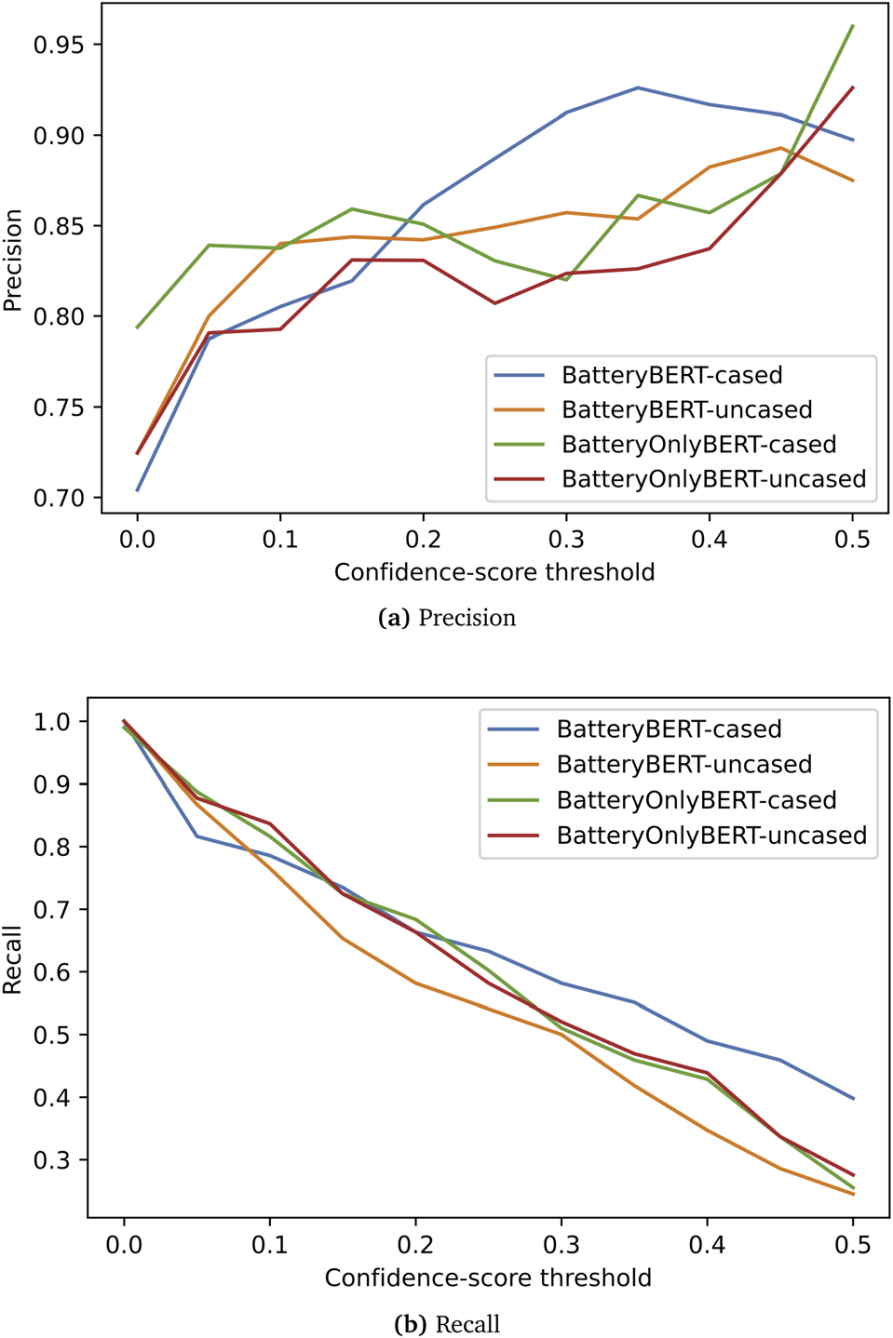
Evaluation results of (a) precision and (b) recall as a function of the confidence-score threshold.

Fig. 5 shows the performance of BatteryDataExtractor on this evaluation data set. Four different previously fine-tuned question-answering models were tested for the data extraction of properties. These models show a similar trend in their precision and recall performance. If no confidence-scorethreshold is used, all data can be extracted, but the precision is only slightly above 70%. However, the precision score increases rapidly when this threshold is employed, as it can filter out the data with a confidence score that is lower than the threshold. Amongst the four models, the BatteryBERT-cased model demonstrates the highest recall when the confidence-score threshold is larger than 0.2, and also the highest precision when using a threshold between 0.2 and ∼0.45. Therefore, the BatteryBERT-cased model was embedded in BatteryDataExtractor for the double-turn question-answering system, given that this model has also been demonstrated to have the best performance on distinguishing types of battery-device data.^44^ Note that when using a confidence-score threshold of 0.1, the precision can be above 80% for most models, while around four-fifths of data still remain in the database. A precision of 80% is comparable to that of the rule-based data-extraction methods that are implemented in ChemDataExtractor,^24^ while BatteryDataExtractor most likely has the capacity to increase the precision score even further by setting a higher threshold. This proves that BatteryDataExtractor has huge potential to bypass rule-based data-extraction methods and auto-generate databases through its embedding of the BatteryBERT model.

## 4 Conclusions

This work has demonstrated the benefits of embedding BERT models into ‘chemistry-aware’ text-mining software for automatically extracting chemical information from scientific documents. The fine-tuned BatteryBERT models outperform the rule-based NLP methods within ChemDataExtractor in terms of its token-classification tasks: abbreviation detection, POS tagging, and CNER. Moreover, by embedding the new fine-tuned double-turn question-answering model into BatteryDataExtractor, the data-extraction pipeline can be switched into another paradigm, where the tedious input of manual rules is no longer required, and inter-related material and property data can be instead implemented with only a few lines of code. Huge deep-learning models such as BatteryBERT can greatly accelerate this text-mining process. Due to the complicated model architecture and the large number of parameters that are necessitated by transformer models, the precision and recall of BERT-based models can remain higher than those implemented by rule-based or ML-based algorithms. In addition to extracting materials-property data, BatteryDataExtractor can also retrieve general information from text of scientific documents by inputting the name of the general information or asking user-defined questions. This function demonstrates the potential power of this approach to create diverse databases from the text in the scientific literature.

One limitation of embedding BERT models into BatteryDataExtractor is that the transformer models are so large, such that multiple high-performance GPUs are required for large-scale data-extraction processes. Several tricks can be helpfully employed in order to improve the efficiency of BatteryDataExtractor, such as knowledge distillation^61^ and quantization.^62^ In addition, while this work has focused on exploring the possibility of applying BatteryBERT models to the area of battery materials, its application can be generalized to a larger domain, such as chemistry and materials science, using different models (*e.g.* MatBERT^42^ and MatSciBERT^43^). As for model performance, the text-mining tasks presented herein can still be improved by using larger deep-learning models or a hybrid system of rule-based and transformer-based algorithms, albeit with a sacrifice of its production efficiency. Lastly, BatteryDataExtractor only processes the raw text data from literature, while the information hidden behind the tables and figures cannot be extracted and analyzed. Table-mining and figure-mining techniques still need to be added in order to retrieve a full literature-mining pipeline.

## Data availability

The source code of BatteryDataExtractor can be found at https://github.com/ShuHuang/batterydataextractor. The documentation of the software is available at https://batterydataextractor.readthedocs.io/. The code used for fine-tuning the BatteryBERT model on the token-classification task can be found at https://github.com/ShuHuang/batterybert/blob/ner/run_ner.py. The fine-tuned token-classification models and evaluation data sets are available at https://huggingface.co/batterydata.

## Author contributions

J. M. C. conceived the overarching project. J. M. C. and S. H. designed the study. S. H. performed the BatteryBERT model fine-tuning for token classification, BatteryDataExtractor software design and development, and data evaluation under the PhD supervision of J. M. C. S. H. drafted the manuscript with assistance from J. M. C.

## Conflicts of interest

There are no conflicts to declare.

## Supplementary Material

SC-013-D2SC04322J-s001
