# Supplementary material for: BatteryDataExtractor: battery-aware text-mining software embedded with BERT models
Source: Chem Sci. 2022 Sep 23;13(39):11487–95. doi: 10.1039/d2sc04322j (PMC9627715; doi:10.1039/d2sc04322j)
Supplement: SC-013-D2SC04322J-s001 [file SC-013-D2SC04322J-s001.zip › SC-013-D2SC04322J-s092.html]

xml version="1.0" encoding="UTF-8"? Structural transformation and electrochemical properties of a nanosized flower-like R-MnO 2 cathode in a sodium battery - Physical Chemistry Chemical Physics (RSC Publishing) DOI:10.1039/D1CP04047B   

View PDF VersionPrevious ArticleNext Article

DOI: 10.1039/D1CP04047B
(Paper)
Phys. Chem. Chem. Phys., 2022, **24**, 551-559

# Structural transformation and electrochemical properties of a nanosized flower-like R-MnO2 cathode in a sodium battery

Kai
Qiu†
a,
Chao
Zhang†
abc,
Mingxia
Yan
a,
Shouwang
Zhao
a,
Hongwei
Fan
a,
Shengli
An
\*a,
Xinping
Qiu
\*b and 

Guixiao
Jia
\*a
  
aSchool of Materials and Metallurgy, Inner Mongolia University of Science and Technology, Baotou, Inner Mongolia 014010, China. E-mail: shengli\_an@126.com; guixiao.jia@163.com
  
bKey Laboratory of Organic Optoelectronics and Molecular Engineering, Department of Chemistry, Tsinghua University, 100084, China. E-mail: qiuxp@mail.tsinghua.edu.cn
  
cSchool of Metallurgical and Ecological Engineering, University of Science and Technology Beijing, 100083, China

Received
3rd September 2021
, Accepted 22nd November 2021

First published on 23rd November 2021

---

## Abstract

High-energy density and low-cost sodium–ion batteries are being sought to meet increasing energy demand. Here, R-MnO2 is chosen as a cathode material of sodium–ion batteries owing to its low cost and high energy density. The structural transformation from the tunnel R-MnO2 to the layered NaMnO2 and electrochemical properties during the charge/discharge are investigated at the atomic level by combining XRD and related electrochemical experiments. Na≤0.04MnO2 has a tunnel R-MnO2 phase structure, Na≥0.42MnO2 has a layered NaMnO2 phase structure, and Na0.04−0.42MnO2 is their mixed phase. Mn3+ 3d4[t2gβ3dz2(1)3dx2−y2(0)] in NaMnO2 loses one 3dz2 electron and the redox couple Mn3+/Mn4+ delivers 206 mA h g−1 during the initial charge. The case that the Fermi energy level difference between R-MnO2 and NaMnO2 is lower than that between the layered Na(12-x)/12MnO2 and NaMnO2 makes the potential plateau of R-MnO2 turning into NaMnO2 lower than that of the layered Na(12−x)/12MnO2 to NaMnO2. This can be confirmed by our experiment from the 1st–2nd voltage capacity profile of R-MnO2 in EC/PC (ethylene carbonate/propylene carbonate) electrolyte. The study would give a new view of the production of sustainable sodium battery cathode materials.

---

## 1. Introduction

Sodium–ion batteries (SIBs) have been studied as low-cost (e.g., the cost of Na2CO3 is about 25–30 times cheaper than Li2CO3)1 and material-abundant alternatives to lithium–ion batteries (LIBs) due to the abundant resource of sodium on earth2 and similarities of intercalation chemistry between SIBs and LIBs.3 However, the larger ionic radius of Na (1.02 Å) than Li (0.76 Å) limits the choice of electrode materials.4 Cathode materials of SIBs such as organic compounds, Prussian blue, polyanionic compounds,5–7 and oxides8–10 have been well investigated. Among all the cathode materials, sodium layered oxides (NaxMO2), in which the structure can be easily regarded as MO2 layers of edge-sharing MO6 octahedron and Na+ occupying interlayer space, have attracted considerable attention.11 Their theoretical capacity is 230–250 mA h g−1 and depends on the M constituents.12 Up to now, there has been a significant level of interest in sodium and manganese based layered oxides, due to their many advantageous properties, such as environmental friendliness and relatively less cost.1 But, sodium layered oxide cathode materials still have some problems such as low average voltage, long-term cycling instability from the effects of Mn3+ Jahn–Teller distortion and multiple phase transitions, and poor rate capability.

A range of different NaxMnO2 (x ≤ 1) structures, from the tunnel to the layered, were synthesized and described by Hagenmuller's research group in 1971.13 Subsequently, a series of NaxMnO2 based materials, Mg-doping compounds (e.g., P2-Na2/3[Mn1−xMgx]O2(0 ≤ x ≤ 0.2)),14 Li-doping compounds (e.g., P2-Na5/6[Mn0.75Li0.25]O2)15 and binary NaxMnTMO2 (TM = Fe, Ni, Co and Cu)16–18 and ternary NaxMnO2 ( = Fe,  = Ni;  = Fe,  = Ti;  = Ni,  = Co;  = Ni,  = Cu; and  = Ni,  = Ti;)19–23 systems and other Mn-based materials for LIBs have been studied.24,25 In these materials Mn3+ 3d4 would give rise to the Jahn–Teller distortion of the Mn3+O6 octahedra, generally leading to structural degradation on cycling.1 For example, MnO2 with α-MnO2, β-MnO2, γ-MnO2, δ-MnO2, λ-MnO2 crystalline phases and so on as the cathode materials of SIBs26–29 suffer the structural instability and Mn2+ dissolution (there is a reaction of Mn3+ → Mn2+ + Mn4+) in electrolyte during the cycling.30–35

Theoretically, MnO2 structural change with various tunnels during the Li+/Na+ ion insertion has been paid attention to. Tompsett and Islam30 found that Li+/Na+ ions distributed themselves as uniformly as possible at the 8h or 8h′ sites of the 2 × 2 tunnel for α-MnO2 and some Mn–O bonds are elongated to change MO6 octahedrons to MO5 tetragonal pyramids. Li et al.36 found that the transformation energy barriers between the layered MnO2 structure and MnO2 with various tunnels are small. However, a distinct picture of the structural transformation from the tunnel to layered MnO2 structures with the ion insertion content is absent, which is important to understand the electrochemical properties of MnO2 during the charge/discharge.

Recently, we prepared nanosized orthorhombic R-MnO2 with the 1 × 2 tunnel structure experimentally and studied its electrochemical properties.37 R-MnO2 is one among various crystalline phases besides α-MnO2, β-MnO2, γ-MnO2, δ-MnO2, and λ-MnO2.37 R-MnO2 transforms from the tunnel to the layered NaMnO2 after initial discharge, and the next discharge/charge is the transformation between the layered Na0.33MnO2 and the layered NaMnO2.37 The initial activation process and the structural change during the charging/discharging are similar to other Mn-based materials, such as Li[Li0.2Ni0.16Mn0.56Co0.08]O2.38 In this work, DFT calculations are carried out to thermodynamically study the structural transformation of MnO2 from the 1 × 2 tunnel to the layered with the Na+ ion insertion content, and XRD experiments are combined to confirm the calculated result. To understand the electrochemical properties, the density of state (DOS), local DOS, and Bader charges of layered NaMnO2 during the charge are investigated at the atomic level. Different potential plateaus of the 1st and 2nd voltage capacity profile in the EC/PC electrolyte from the experiment are explained by exploring Fermi energy level and electron density distribution of the highest occupied molecular orbital (HOMO) of R-MnO2 and the layered Na(12−x)/12MnO2.

## 2. Calculation method and model

R-MnO2 with the 1 × 2 tunnel structure is orthorhombic (Space group: Pnma), and optimized primitive lattice parameters are a = 9.51 Å (9.27 Å), b = 2.93 Å (2.86 Å), c = 4.60 Å (4.52 Å) (The values in the parenthesis are from the experiment39). Layered NaMnO2 is monoclinic (space group: C2/m) and optimized primitive lattice parameters a = 5.74 Å (5.67 Å), b = 2.92 Å (2.86 Å), c = 5.81 Å (5.81 Å) and β = 113.55°(113.2°) (The values in the parenthesis are from the experiment40). 2a × 1b × 3c R-MnO2 (Mn24O48) (Fig. 1a) and 4a × 1b × 3c layered NaMnO2 supercells (Na24Mn24O48) (Fig. 1b) are selected to explore the structural transformation of R-MnO2 from the tunnel to the layered. To shorten the calculation time, the layered Na12−xMn12O24 (0 ≤ x ≤ 12) system containing 48 atomic positions with a 1a × 3b × 2c supercell (Fig. 1c) is considered to study the electronic structures during charging.
  

|  |  |  |
| --- | --- | --- |
|  | | |
|  | **Fig. 1**  The crystal structure of the 2a × 1b × 3c R-MnO2 (a), monoclinic layered NaMnO2 structures with a 4a × 1b × 3c supercell (b) and a 1a × 3b × 2c supercell (c), the SEM image of R-MnO2 (d), and XRD powder patterns (e) for layered NaMnO2 and Na0.33MnO2 electrode after initial charge and discharge (solid lines) as well as calculated NaMnO2 and Na0.33MnO2 (dots). |  |

All the DFT calculations were performed using the Vienna Ab Initio Simulation package (VASP) within a projector augmented-wave (PAW) pseudo potential approach. A spin-polarized generalized gradient approximation (GGA) with the Perdew–Burke–Ernzerhof (PBE)41 exchange–correlation functional was employed. The electronic structures, Na 3s13p0, Mn 3d64s1 and O 2s22p4, were selected. Considering that the element Mn has a higher angular quantum number of 3d electrons, we applied the GGA+U method described using the parameters U and J. The Hubbard U and J values of Mn are 6.0 eV and 1.0 eV, respectively, similar to the simplified U–J value of our previous42 and other works.43 The energy cutoff for the plane waves was set to 520 eV. Monkhorst–Pack scheme 1 × 6 × 2 k-points meshes for 2a × 1b × 3c R-MnO2 (Fig. 1a) and 4a × 1b × 3c layered NaMnO2 supercells, and Monkhorst–Pack scheme 4 × 2 × 2 k-points meshes for 1a × 3b × 2c layered Na12−xMn12O24 (0 ≤ x ≤ 12) were used for ion relaxations, lattice parameter optimization, and electronic structures. Atomic structures were fully optimized until forces acting on every atom became at least smaller than 0.02 eV Å−1, and the total energy was converged to 10−4 eV.

The SEM image of R-MnO2 is given in Fig. 1d. Secondary particles of R-MnO2 show flower-like spherical morphology with a diameter of 1.7 μm, which is composed of rod-like crystallites with a width of about 50 nm. Simulated XRD from layered NaMnO2 and Na0.33MnO2 fits with that of the initial discharging (NaMnO2) and charging (Na0.33MnO2) material, respectively (Fig. 1e). These primary particles of R-MnO2 less than 100 nm lead to the appearance of a large peak shape distribution. There appears a characteristic peak at 17° and a broad peak at 35–40°.

The phase structural transformation of MnO2 from the tunnel to the layered under the different sodium contents is studied thermodynamically. The energy difference between both structures is expressed by ΔE:

|| ΔE = (E1 − E2)/2x | (1) |

E1 and E2 are the energies of Na2xMn24O48 (0 ≤ x ≤ 12) systems with R-MnO2 and layered NaMnO2 structures, respectively. 2x is the number of Na atoms. When the absolute value of ΔE is obviously larger than 0, the tunnel structure or the layered structure is stable. However, when the absolute value of ΔE is close to 0, two structures might coexist, meaning that there are two phases during the first cycle discharge of R-MnO2.

In order to understand the structural stability of layered Na12−xMn12O24 (0 ≤ x ≤ 12) during the charge, the enthalpy change ΔH was calculated, and the corresponding formula was followed,

||  | (2) |

here, E(Na12−xMn12O24), E(Na12Mn12O24), and E(Mn12O24) are the total energies of layered Na12−xMn12O24, layered Na12Mn12O24, and layered Mn12O24 systems, respectively. x is the Na+ ion de-intercalation content. Structural distortion of layered NaMnO2 derived from the continuous charge was described using distortion energies Ed:

||  | (3) |

here, E(Mn12O24 − Na12−xM12O24) and E(Mn12O24 − Na12M12O24) are the total energies of Mn12O24 removing all Na+ ions from the layered Na12−xMn12O24 and layered Na12Mn12O24 systems, respectively.

## 3. Results and discussion

### 3.1. Geometrical structures and stabilities

Our experiment37 found that the orthorhombic tunnel R-MnO2 was transformed to the layered monoclinic NaMnO2 during the first cycle discharge, then, the layered NaMnO2 structure is retained, and the Na0.33MnO2 component is formed during the next cycle charge. In all, the change between the layered NaMnO2 structure and the layered Na0.33MnO2 structure during the later cycle charge/discharge occurs. Therefore, in this work, the structural changes of R-MnO2 during the discharge and layered NaMnO2 during the charge and the structural transformation of MnO2 from the tunnel to the layered are explored thermodynamically.

3.1.1. Structural change of R-MnO2 during Na+ ion insertion. 
Various possible sites of Na+ ion insertion to orthorhombic R-MnO2 are considered, and the system with the 4c (Fig. 2a) site has the lowest energy. When the number of Na+ ions inserted to Mn24O48 is more than 3, effects of mutual repulsion between Na+ ions open the 1 × 2 tunnels (Fig. 2c–e) until all 1 × 2 tunnels are opened as the NaMnO2 component is formed (Fig. 2f). Li et al.36 found that a common shearing and buckling movement of the MnO2 layer led to the breaking of the Mn–O framework when the layered MnO2 structure was transformed to MnO2 ones with 1 × 1, 1 × 2, and 2 × 2 tunnels. In the same way, the breaking of the Mn–O framework will also occur when the MnO2 structures with 1 × 1, 1 × 2, and 2 × 2 tunnels are transformed to the layered MnO2 during Na+ ion insertion. The behavior similarly happens at the structural change of R-MnO2 during Na+ ion insertion, which is consistent with Li's result.36
  

|  |  |  |
| --- | --- | --- |
|  | | |
|  | **Fig. 2**  The tunnel Na2xMn24O48 structures at 2x = 1 (a), 3 (b), 4 (c), 8 (d), 20 (e), and 24 (f). |  |

3.1.2. Structures and stabilities of layered Na12−xMn12O24 during Na+ ion de-intercalation. 
Stable geometrical structures of layered Na12−xMn12O24 after the Na+ ion de-intercalation are obtained (Fig. 3). The de-intercalation rule of Na+ ions is to ensure that the remaining Na+ ions are uniformly dispersed. The lattice parameters are shown in Table 1.
  

|  |  |  |
| --- | --- | --- |
|  | | |
|  | **Fig. 3**  Structures of Na12−xMn12O24 at x = 0 (a), 4 (b), and 8 (c) from the view point of the Na layer. ( Na  O  Mn). |  |

**Table 1** Calculated lattice parameters of the unit cell, the O–O layer spacing (d), volume contraction (ΔV), distortion energies (Ed), and the enthalpy change (ΔH) of Na12−xMn12O24

| x in Na12−xMn12O24 | a (Å) | b (Å) | c (Å) | B (°) | d (Å) | ΔV (%) | E d (eV) | ΔH (eV) |
| --- | --- | --- | --- | --- | --- | --- | --- | --- |
| 0 | 5.74 | 2.92 | 5.81 | 113.55 | 3.20 | — | — | 0.07 |
| 1 | 5.64 | 2.93 | 5.80 | 112.45 | 3.68 | 0.90 | 0.0 | −0.23 |
| 2 | 5.57 | 2.92 | 5.80 | 111.73 | 3.70 | 1.89 | −0.62 | −0.80 |
| 3 | 5.48 | 2.93 | 5.79 | 110.67 | 3.69 | 2.81 | −1.17 | −0.95 |
| 4 | 5.47 | 2.91 | 5.81 | 110.37 | 3.73 | 3.04 | −0.95 | −1.87 |
| 5 | 5.38 | 2.92 | 5.83 | 109.30 | 3.79 | 3.31 | −1.05 | −1.86 |
| 6 | 5.32 | 2.91 | 5.87 | 108.68 | 3.91 | 3.46 | −1.01 | −2.20 |
| 7 | 5.24 | 2.92 | 5.89 | 107.68 | 3.98 | 3.69 | −1.04 | −2.08 |
| 8 | 5.24 | 2.91 | 5.95 | 107.82 | 3.74 | 3.44 | −0.91 | −2.47 |
| 10 | 5.15 | 2.93 | 5.92 | 107.52 | 3.90 | 4.80 | −0.88 | −1.14 |
| 12 | 5.09 | 2.94 | 5.14 | 105.80 | 3.17 | 17.06 | −0.80 | 0.0 |

---

  

From Table 1, it can be seen that β angle decreases as x increases. Lattice constant a takes on an obvious decreasing trend. Lattice constant c slightly increases. Lattice constant b is almost invariable. Due to the mutual Coulomb repulsion of oxygen ions, O–O layer spacing d between two different MnO2 layers in Na12−xMn12O24 increases in general as x increases. The unit cell volume contraction ΔV of Na12−xMn12O24 compared to Na12Mn12O24 increases with x. When all Na ions are removed, ΔV of 17.06% would be adverse, and the lattice c and the O–O layer spacing d for the system at x = 12 becomes small. Except for it, the largest value of the volume swell is only 4.80%. The changing features of volume contraction ΔV and the O–O layer spacing d in Na0.33MnO2 (Na12−xMn12O24 at x = 8) are fluctuating, and it is interesting that ΔH of Na0.33MnO2 is the smallest, see Table 1.

To examine the thermodynamic stability of Na12−xMn12O24 during the Na+ ion de-intercalation, the enthalpy change ΔH is calculated (Fig. 4a and Table 1). From the obtained ΔH, ΔH of Na12−xMn12O24 at x = 8 is the lowest, indicating that the sodium-deficient phase could be experimentally synthesized. The distortion energies Ed derived from the Na+ ion de-intercalation are negative because of the shrinkage of Mn–O bonds and trend to a constant before x ≥ 3 (Fig. 4a and Table 1). In the layered NaMnO2, Mn3+ with the d4 electron structure leads to Jahn–Teller distortion of MnO6 octahedron, so there are two types of Mn–O bond length: short bonds A and long bonds B. As the de-intercalation amount x in Na12−xMn12O24 increases, there are more and more Mn4+ until all Mn3+ are oxidized to Mn4+ at x = 12, so long bonds B shorten and their distribution range gradually converges to that of a short bond with 1.947 Å (Fig. 4b).

  

|  |  |  |
| --- | --- | --- |
|  | | |
|  | **Fig. 4**  The enthalpy change ΔH and distortion energies Ed (a) and Mn–O bond length (b) as x in Na12−xMn12O24. |  |

3.1.3. Structural transformation of R-MnO2 to layered NaMnO2. 
The phase structural transformation of MnO2 from the tunnel to the layered under the different sodium contents is studied thermodynamically. The energy difference ΔE is plotted in Fig. 5a. The absolute value of ΔE is obviously larger than zero at 2x ≤ 1 (Na0.04MnO2) and 2x ≥ 10 (Na0.42MnO2) in Na2xMn24O48 and the former ΔE is negative, and the latter ΔE is positive; however, the absolute value of ΔE is close to zero at 1 < 2x < 10. This means that Na≤0.04MnO2 is inclined to be the tunnel structure, Na≥0.42−1MnO2 is the layered structure, and Na0.04−0.42MnO2 is their mixed phase structure. The calculated result can be confirmed by the XRD experiment. The XRD powder pattern of discharged R-MnO2 electrode is shown in Fig. 5b. It can be seen that the characteristic peaks of the R-MnO2 phase at 2θ = 22.4° are gradually decreased as the sodium content increases. In Na0.03MnO2, there is a characteristic peak at 2θ = 22.4° and no characteristic peak at 2θ = 16.6°, and thus, Na0.03MnO2 is the R-MnO2 phase with the tunnel structure. In Na0.2MnO2, the characteristic peak at 2θ = 16.6° appears, namely the layered monoclinic NaMnO2 phase is formed. In Na0.5MnO2, the characteristic peaks of the R-MnO2 phase at 2θ = 22.4° disappear, while the intensity of the characteristic peak with layered NaMnO2 structure phase at 2θ = 16.6° increases, showing that only the layered NaMnO2 phase exists in the electrode. The Na0.2−0.5MnO2 would be a mixed phase with the tunnel and the layered structures.
  

|  |  |  |
| --- | --- | --- |
|  | | |
|  | **Fig. 5**  Energy difference ΔE of Na2xMn24O48 between R-MnO2 and layered NaMnO2 structures (a) and XRD powder patterns of Na0.03MnO2, Na0.2MnO2, Na0.33MnO2, Na0.5MnO2, Na0.6MnO2, and R-MnO2 (expressed as initial) (b). The insert picture in (a) is O3-type phase structure of Na0.33MnO2, and share edges MnO6 are denoted as “E”. Two XRD powder patterns at the bottom of (b) are those of R-MnO2 and layered monoclinic NaMnO2, respectively. |  |

The layered Na0.33MnO2 has an O3-type monoclinic structure. It can be transformed from O′3-type phase structure by gliding of the MnO2 slab without the breakage of Mn–O bonds. The characteristic of NaO6 octahedron, sharing only edges in the O′3-type phase structure, also exists in O3-type, as shown in Fig. 5a.

### 3.2. Redox process of layered Na12−xMn12O24

The density of state (DOS) and partial DOS (PDOS) directly reflecting the electronic structural change are calculated to understand the redox process of Mn in the layered Na12−xMn12O24 system (Fig. 6). NaMnO2 has a band gap of about 0.6 eV. It varies from being a semiconductor to a conductor with the sodium de-intercalation, and finally, the band gap is opened as all Na+ ions are removed (Fig. 6a). The Jahn–Teller distortion of Mn3+ leads to a split of two eg orbits, among which dz2 orbital is occupied at −1.0 to – 0 eV and dx2−y2 orbital is empty, as shown in Fig. 6b. In PDOS of Na12−xMn12O24, all up-spin states of t2g and one up-spin state of  are occupied, and another up-spin state of  and both down-spin states of t2g and  are unoccupied in Na12-xMn12O24 at x = 0, inferring Mn ions are in the +3 state. With the sodium de-intercalation, more and more up-spin dz2 states are unoccupied, indicating the Mn4+ ion formation (Fig. 6b). This can be confirmed by the XPS spectra of the R-MnO2 electrode after the 2nd charge.37 In the XPS spectra, Mn3+ and Mn4+ characteristic peaks are located at ∼640.5 eV and ∼641.8 eV, respectively, as in our previous work.37 Oxidization of Mn3+ in Na12−xMn12O24 is also supported by Bader charges, as shown in Table 2. The Bader charge of Mn3+ is 5.358 e − 5.411 e, and the Bader charge of Mn4+ is 5.272 e − 5.351 e.
  

|  |  |  |
| --- | --- | --- |
|  | | |
|  | **Fig. 6**  Evolution of TDOS (a) and PDOS of Mn3+/Mn4+ (b) for Na12−xMn12O24. |  |

**Table 2** Bader charges of Mn4+ and Mn3+ in layered Na12−xMn12O24. The number in parenthesis is that of Mn4+ or Mn3+

| x | 0 | 1 | 2 | 3 | 4 | 5 | 6 | 7 | 8 | 10 | 12 |
| --- | --- | --- | --- | --- | --- | --- | --- | --- | --- | --- | --- |
| Mn4+ | — | 5.351 (1) | 5.310 (2) | 5.315 (3) | 5.288 (4) | 5.302 (5) | 5.289 (6) | 5.295 (7) | 5.287 (8) | 5.277 (10) | 5.272 (12) |
| Mn3+ | 5.411 (12) | 5.405 (11) | 5.399 (10) | 5.389 (9) | 5.389 (8) | 5.379 (7) | 5.375 (6) | 5.369 (5) | 5.361 (4) | 5.358 (2) | — |

---

  

In order to visually understand the Mn4+ ion formation, the local charge density of Mn ions in the Na0.33MnO2 system located at −1.0 to –0 eV is presented, see Fig. 7. From Fig. 7a, it can be seen that electron density only focuses on 4 Mn ions among 12 Mn ions (0.33 ratio), namely 0.67 Mn3+ ions in Na0.33MnO2 are oxidized to Mn4+. We can also see that Mn3+ 3dz2 and O 2pz form a σ\* anti-bond (The corresponding Mn3+–O bond length is 2.214 Å, see Fig. 8d in the following part), consistent with the charge density difference result (Fig. 7b). In Fig. 7b, the Mn4+–O bond is obviously ionic, while the Mn3+–O bond has anti-bonding characteristics due to charge repulsion between the positive charge density from Mn3+ and O2−.

  

|  |  |  |
| --- | --- | --- |
|  | | |
|  | **Fig. 7**  Charge density at –1.0 to –0 eV (a) and the right of (a) is the figure of the interaction between Mn3+ and O2− ions. Charge density difference of Na0.33MnO2 (b) and the right of (b) gives a slice of charge density difference. |  |

  

|  |  |  |
| --- | --- | --- |
|  | | |
|  | **Fig. 8**  The 1st–2nd voltage capacity profile of R-MnO2 in the EC/PC electrolyte (a). The relative Fermi energy level of layered Na12−xMnO2 and R-MnO2 (b), where the Fermi level of NaMnO2 is set to 0. Energy band structures and HOMO of NaMnO2 (c), Na0.33MnO2 (d), and R-MnO2 (e). Here, Mn–O bond lengths of one MnO6 octahedron are given, and the unit is Å. |  |

### 3.3. Potential plateau during the discharge

From our experiment,37 it is known that R-MnO2 forms the layered NaMnO2 after the initial discharge, NaMnO2 retains its layered structure after the initial charge, and the next discharge/charge is the transformation between the layered Na0.33MnO2 and the layered NaMnO2. The initial activation process and the structural change during the charging/discharging are similar to other Mn-based materials such as Li[Li0.2Ni0.16Mn0.56Co0.08]O2.38 Potential plateaus of R-MnO2 turning into the layered NaMnO2 and the layered Na0.33MnO2 turning into the layered NaMnO2 are different (Fig. 8a). In addition, the complete charge–discharge curve and cycling performance have been taken in our previous work using BMIMTFSI and EC/PC electrolytes.37 In order to explain the phenomenon, Fermi energy levels of various layered Na12−xMn12O24 compositions and R-MnO2 are calculated (Fig. 8b). The aim is to use two differences between Fermi energy levels of Na12−xMn12O24 and NaMnO2, and R-MnO2 and NaMnO2 to reflect two different potential plateaus of transformations from Na12−xMn12O24 to NaMnO2 and R-MnO2 to NaMnO2 during the discharge. Fermi energy of one bulk system can be obtained by calculating the Fermi energy of its most stable surface or its first ionization potential (IP). Our work selects the second method. IP is the difference between total energies of the neutral substance and the system with one electron removed to infinity, expressed as

|| IP = E(+) − E(0) | (4) |

E(+) and E(0) are the energies per Na(12−x)/12MnO2 formula with and without one positive charge, respectively. In Fig. 8b, the Fermi energy level of NaMnO2 is the lowest, it lifts with the increase in x of Na12−xMn12O24, and the Fermi energy levels of R-MnO2 and Na11Mn12O24 are close. The Fermi energy difference of R-MnO2 to NaMnO2 is lower than that of Na(12−x)/12MnO2 to NaMnO2. So, the phenomenon that the potential plateau of R-MnO2 turning into the layered NaMnO2 is lower than that of the layered Na0.33MnO2 transforming to the layered NaMnO2 is seen in our experiment result.

The HOMO and the geometrical structures of the layered Na0.33MnO2, layered NaMnO2, and R-MnO2 are employed to understand their Fermi energy level variation. To obtain HOMO, energy band structures of these systems are first calculated to get the highest occupied energy level at a certain k-point, and subsequently, the electronic structure at the energy level, namely HOMO, is computed (Fig. 8c–e). The MnO6 octahedron of NaMnO2 has two long Mn–O bonds (2.428 Å) and four short Mn–O bonds (1.969 Å) due to the Jahn–Teller distortion of Mn3+3d4. In HOMO of NaMnO2, the interaction between O 2p and Mn 3d in four short Mn–O bonds is non-bonding. There is a similar case between O 2p and Mn 3d in three shorter Mn–O bonds (1.843 Å, 1.843 Å, and 1.871 Å) for R-MnO2. In NaMnO2, the Jahn–Teller distortion of Mn3+ 3d4 would benefit the structural stability. So, the Fermi energy level of NaMnO2 is lower than that of R-MnO2. The Na0.33MnO2 system has Mn3+ and Mn4+. The interaction between O 2p and Mn 3d in the long Mn3+–O bonds (2.214 Å) is anti-bonding because the direction of O 2p orbital and Mn3+ 3d orbital is in line. This would lead to a higher Fermi energy level of Na0.33MnO2 than NaMnO2 and R-MnO2 systems. The interaction between O 2p and Mn 3d in HOMO of other Na(12−x)/12MnO2 systems have the same anti-bonding characteristic to Na0.33MnO2, resulting in a whole rule that the Fermi energy level difference between R-MnO2 and NaMnO2 is lower than that between Na(12−x)/12MnO2 and NaMnO2.

## 4. Conclusion

In this work, nanosized flower-like R-MnO2 with a 1 × 2 tunnel prepared experimentally is discharged to obtain a monoclinic layered NaMnO2 cathode material. The next cycle charge/discharge transformation happens between the two layered structures of NaMnO2 and Na0.33MnO2. During the charging process, the cathode material delivers 206 MA h g−1 through the redox couple of Mn3+/Mn4+. R-MnO2 retains its structure when the molar content of inserted Na does not exceed the value of 0.04. It is transformed into the layered NaMnO2 structure when the insertion Na molar content is ≥0.42. The middle composition is the mixed phase of the tunnel and the layered structures. The different potential plateaus of R-MnO2 turning into the layered NaMnO2 and the layered Na0.33MnO2 transforming to the layered NaMnO2 are from the distinctions of two Fermi level differences between R-MnO2/NaMnO2 and Na0.33MnO2/NaMnO2. The work not only provides new insight into a deeper understanding of the redox chemistry in sodium ion cathode materials, but also facilitates the development of higher capacity electrons for sodium ion batteries.

## Conflicts of interest

There are no conflicts to declare.

## Acknowledgements

The authors appreciate financial support from China-US Electric Vehicle Project (S2016G9004), National Key Project on Fundamental Research Program (2015CB251104), and Inner Mongolia Natural Science Foundation (No. 2021MS02003).


## References

1. N. OrtizVitoriano, E. Drewett, E. Gonzaloa and T. Rojo, High performance manganese-based layered oxide cathodes: overcoming the challenges of sodium ion batteries., Energy Environ. Sci., 2017, 10, 1051–1074 RSC.
2. S. Kim, D. H. Seo, X. Ma, G. Ceder and K. Kang, Electrode Materials for Rechargeable Sodium–Ion Batteries: Potential Alternatives to Current Lithium–Ion Batteries, Adv. Energy. Mater., 2012, 2, 710–721 CrossRef CAS.
3. R. S. Carmichael, Practical handbook of physical properties of rocks and minerals, CRC Press, 1989 Search PubMed.
4. D. P. K. Nayak, L. Yang, W. Brehm and P. Adelhelm, From Lithium–Ion to Sodium–Ion Batteries: Advantages, Challenges, and Surprises, Angew. Chem., Int. Ed., 2018, 57, 102 CrossRef PubMed.
5. J. Zhang, Y. Fang, L. Xiao, J. Qian, Y. Cao, X. Ai and H. Yang, From Lithium–Ion to Sodium–Ion Batteries: Advantages, Challenges, and Surprises, ACS Appl. Mater. Interfaces, 2017, 9, 7177 CrossRef CAS PubMed.
6. X. Wu, G. Zhong and Y. Yang, Sol–gel synthesis of Na4Fe3(PO4)2(P2O7)/C nanocomposite for sodium ion batteries and new insights into microstructural evolution during sodium extraction, J. Power Sources, 2016, 327, 666–674 CrossRef CAS.
7. Z. Jian, L. Zhao, H. Pan, Y. S. Hu, H. Li, W. Chen and L. Chen, Carbon coated Na3V2(PO4)3 as novel electrode material for sodium ion batteries, Electrochem. Commun., 2012, 14, 86–89 CrossRef CAS.
8. Z. Chen, T. Yuan., X. Pu, H. Yang, X. Ai, Y. Xia and Y. Cao, Symmetric Sodium–Ion Capacitor Based on Na0.44MnO2 Nanorods for Low-Cost and High-Performance Energy Storage, ACS Appl. Mater. Interfaces, 2018, 10, 11689–11698 CrossRef CAS PubMed.
9. M. Xie, R. Luo, J. Lu, R. Chen, F. Wu, X. Wang, C. Zhan, H. Wu, H. M. Albishri and A. S. Al-Bogami, Synthesis-Microstructure-Performance Relationship of Layered Transition Metal Oxides as Cathode for Rechargeable Sodium Batteries Prepared by High-Temperature Calcination, ACS Appl. Mater. Interfaces, 2014, 6, 17176–17183 CrossRef CAS PubMed.
10. K. Kubota, N. Yabuuchi, H. Yoshida, M. Dahbi and S. Komaba, Layered oxides as positive electrode materials for Na-ion batteries, MRS Bull., 2014, 39, 416–422 CrossRef CAS.
11. M. H. Han, E. Gonzalo, G. Singh and T. Rojo., A comprehensive review of sodium layered oxides: powerful cathodes for Na-ion batteries, Energy Environ. Sci., 2015, 8, 81–102 RSC.
12. N. Yabuuchi, M. Kajiyama, J. Iwatate, H. Nishikawa, S. Hitomi, R. Okuyama, R. Usui, Y. Yamada and S. Komaba, P2-type Nax[Fe1/2Mn1/2]O2 made from earth-abundant elements for rechargeable Na batteries, Nat. Mater., 2012, 11, 512–517 CrossRef CAS PubMed.
13. J. P. Parant, R. Olazcuaga and M. Devalette, Sur Quelques Nouvelles Phases de Formule NaxMnO2(x = 1), J. Solid State Chem., 1971, 3, 1–11 CrossRef CAS.
14. J. Billaud, G. Singh, A. R. Armstrong, E. Gonzalo, V. Roddatis, M. Armand, T. Rojo and P. G. Bruce, Na0.67Mn1−xMgxO2 (0 ≤ x ≤ 0.2): a high capacity cathode for sodium-ion batteries, Energy Environ. Sci., 2014, 7, 1387–1391 RSC.
15. N. Sharma, N. Tapia-Ruiz, G. Singh, A. R. Armstrong, J. C. Pramudita, H. E. A. Brand, J. Billaud, P. G. Bruce and T. Rojo, Rate Dependent Performance Related to Crystal Structure Evolution of Na0.67Mn0.8Mg0.2O2 in a Sodium–Ion Battery, Chem. Mater., 2015, 27, 6976–6986 CrossRef CAS.
16. J. S. Thorne., R. A. Dunlap and M. N. Obrovac, A Combinatorial study of the Sn–Si–C system for Li-ion battery applications, J. Electrochem. Soc., 2012, 160, A361–A367 CrossRef.
17. X. Wang, M. Tamaru, M. Okubo and A. Yamada, Electrode Properties of P2-Na2/3MnyCo1−yO2 as Cathode Materials for Sodium–Ion Batteries, J. Phys. Chem. C, 2013, 117, 15545–15551 CrossRef CAS.
18. Z. Lu and J. R. Dahn, Situ X-Ray Diffraction Study of P2 Na2/3[Ni1/3Mn2/3]O2, J. Electrochem. Soc., 2001, 148, A1225–A1229 CrossRef CAS.
19. I. Hasa, D. Buchholz, S. Passerini, B. Scrosati and J. Hassoun, High Performance Na0.5[Ni0.23Fe0.13Mn0.63]O2 Cathode for Sodium-Ion Batteries, Adv. Energy Mater., 2014, 4, 1400083 CrossRef.
20. M. H. Han, E. Gonzalo, N. Sharma, J. M. Lopez Del Amo, M. Armand, M. Avdeev, J. J. Saiz, J. J. S. Garitaonandia and T. Rojo, High-Performance P2-Phase Na2/3Mn0.8Fe0.1Ti0.1O2 Cathode Material for Ambient-Temperature Sodium-Ion Batterie, Chem. Mater., 2016, 28, 106–116 CrossRef CAS.
21. D. Yuan, W. He, F. Pei, F. Wu, Y. Wu, J. Qian, Y. Cao, X. Ai and H. Yang, Synthesis and electrochemical behaviors of layered Na0.67[Mn0.65Co0.2Ni0.15]O2 microflakes as a stable cathode material for sodium-ion batteries, J. Mater. Chem. A, 2013, 1, 3895–3899 RSC.
22. L. Zheng, J. Li and M. N. Obrovac, Crystal Structures and Electrochemical Performance of Air-Stable Na2/3Ni1/3−x,CuxMn2/3O2 in Sodium Cells, Chem. Mater., 2017, 29, 1623–1631 CrossRef CAS.
23. H. Yoshida, N. Yabuuchi, K. Kubota, I. Ikeuchi, A. Garsuch, M. Schulz-Dobrick and S. Komaba, P2-type Na2/3Ni1/3Mn2/3−xTixO2 as a new positive electrode for higher energy Na-ion batteries, Chem. Commun., 2014, 50, 3677–3680 RSC.
24. X. H. Zhu, F. Q. Meng, Q. H. Zhang, L. Xue, H. Zhu, S. Lan, Q. Liu, J. Zhao, Y. H. Zhuang, Q. B. Guo, B. Liu, L. Gu, X. Lu, Y. Ren and H. Xia, LiMnO2 Cathode Stabilized by Interfacial Orbital Ordering for Sustainable Lithium-Ion Batteries, Nat. Sustain., 2021, 4, 392–401 CrossRef.
25. X. H. Zhu, Q. Y. Xia, X. Y. Liu, Q. H. Zhang, J. Xu, B. W. Lin, S. Li, Y. H. Zhuang, C. Qiu, L. Xue, L. Gu and H. Xia, Retarded Layered-to-Spinel Phase Transition in Structure Reinforced Birnessite with High Li Content, Sci. Bull., 2021, 66, 219–224 CrossRef CAS.
26. N. Wang, H. Pang, H. Peng, G. Li and X. Chen, Hydrothermal synthesis and electrochemical properties of MnO2 nanostructures, Cryst. Res. Technol., 2009, 44, 1230–1234 CrossRef CAS.
27. C. M. Julien and A. Mauger, Nanostructured MnO2 as Electrode Materials for Energy Storage, Nanomaterials, 2017, 7, 396 CrossRef PubMed.
28. Y. Zhou, T. Chen, J. Zhang, Y. Liu and P. Ren, Amorphous MnO2 as Cathode Material for Sodium-ion Batteries, Chin. J. Chem., 2017, 35, 1294–1298 CrossRef CAS.
29. D. Lv, X. Huang, H. Yue and Y. Yang, Sodium-Ion-Assisted Hydrothermal Synthesis of gamma-MnO2 and Its Electrochemical Performance, J. Electrochem. Soc., 2009, 156, A911–A916 CrossRef CAS.
30. A. T. David. and M. S. Islam, Electrochemistry of Hollandite alpha-MnO2: Li–Ion and Na–Ion Insertion and Li2O Incorporation, Chem. Mater., 2013, 25, 2515–2526 CrossRef.
31. Y. An, J. Feng, L. Ci and S. Xiong, MnO2 nanotubes with a water soluble binder as high performance sodium storage materials, RSC Adv., 2016, 6, 103579 RSC.
32. B. Kishore, G. Venkatesh and N. Munichandraiah, A Na/MnO2 Primary Cell Employing Poorly Crystalline MnO2, J. Electrochem. Soc., 2015, 162, A839–A844 CrossRef CAS.
33. D. Su, H. J. Ahn and G. Wang, Beta-MnO2 nanorods with exposed tunnel structures as high-performance cathode materials for sodium-ion batteries, NPG. Asia Mater., 2013, 5, e70 CrossRef CAS.
34. D. Su, H. J. Ahn and G. Wang, Hydrothermal synthesis of alpha-MnO2 and beta-MnO2 nanorods as high capacity cathode materials for sodium ion batteries, J. Mater. Chem. A, 2013, 1, 4845 RSC.
35. Y. Liu, Y. Qiao, W. Zhang, H. Wang, K. Chen, H. Zhu, Z. Li and Y. Huang, Nanostructured alkali cation incorporated delta-MnO2 cathode materials for aqueous sodium-ion batteries, J. Mater. Chem. A, 2015, 3, 7780–7785 RSC.
36. Y. F. Li, S. C. Zhu and Z. P. Liu, Reaction Network of Layer-to-Tunnel Transition of MnO2, J. Am. Chem. Soc., 2016, 138, 5371–5379 CrossRef CAS PubMed.
37. H. N. Si, L. Li, W. J. Hao, L. Seidl, X. L. Cheng, H. Y. Xu, G. X. Jia, O. Schneider, S. L. An and X. P. Qiu, Structural Transformation and Cycling Improvement of Nanosized Flower-like γ-MnO2 in a Sodium Battery, ACS. Appl. Energy. Mater., 2019, 2, 5050–5056 CrossRef CAS.
38. D. Buchholz, J. Li, S. Passerini, G. Aquilanti, D. D. Wang and M. Giorgetti, X-Ray Absorption Spectroscopy Investigation of Lithium-Rich, Cobalt-Poor Layered-Oxide Cathode Material with High Capacity, ChemElecctroChem., 2015, 2, 85–97 CrossRef CAS.
39. J. E. Post and P. J. Heaney, Neutron and synchrotron X-ray diffraction study of the structures and dehydration behaviors of ramsdellite and “groutellite”, Am. Mineral., 2004, 89, 969–975 CrossRef CAS.
40. O. I. Velikokhatnyi, D. Choi and P. N. Kumta, Energetics of the lithium-magnesium imide–magnesium amide and lithium hydride reaction for hydrogen storage: An ab initio study, Mater. Sci. Eng., B, 2006, 128, 115–124 CrossRef CAS.
41. J. P. Perdew, K. Burke and M. Ernzerhof, Generalized gradient approximation made simple, Phys. Rev. Lett., 1996, 77, 3865–3868 CrossRef CAS PubMed.
42. H. Z. Wei, X. L. Cheng, H. W. Fan, Q. Shan, S. L. An, X. P. Qiu and G. X. Jia, A Cobalt-Free Li(Li0.17Ni0.17Fe0.17Mn0.49)O2 Cathode with More Oxygen-Involving Charge Compensation for Lithium-Ion Batteries, ChemSusChem, 2019, 12, 2471–2479 CAS.
43. Z. Z. Zhang, Y. F. Zhang, Y. Li, J. Lin, D. G. Truhlar and S. P. Huang, MnSb2S4 Monolayer as an Anode Material for Metal-Ion Batteries, Chem. Mater., 2018, 30, 3028–3214 CrossRef.

---

|  |
| --- |
| Footnote |
| † These authors contribute equally to this work. |

|  |
| --- |
| --- |
| **This journal is © the Owner Societies 2022** |

 
