# Supplementary material for: BatteryDataExtractor: battery-aware text-mining software embedded with BERT models
Source: Chem Sci. 2022 Sep 23;13(39):11487–95. doi: 10.1039/d2sc04322j (PMC9627715; doi:10.1039/d2sc04322j)
Supplement: SC-013-D2SC04322J-s001 [file SC-013-D2SC04322J-s001.zip › SC-013-D2SC04322J-s093.html]

xml version="1.0" encoding="UTF-8"? Surface modification of electrospun TiO 2 nanofibers via layer-by-layer self-assembly for high-performance lithium-ion batteries - Journal of Materials Chemistry (RSC Publishing) DOI:10.1039/C2JM15197A   

View PDF VersionPrevious ArticleNext Article

DOI: 10.1039/C2JM15197A
(Paper)
J. Mater. Chem., 2012, **22**, 4910-4915

Show CompoundsShow Chemical Terms

# Surface modification of electrospun TiO2 nanofibers via layer-by-layer self-assembly for high-performance lithium-ion batteries†

Wei
Luo
,
Xianluo
Hu
\*,
Yongming
Sun
 and 

Yunhui
Huang
\*
  
State Key Laboratory of Material Processing and Die & Mould Technology, School of Materials Science and Engineering, Huazhong University of Science and Technology, Wuhan, 430074, P. R. China. E-mail: huxl@mail.hust.edu.cn; xlhu07@gmail.com; huangyh@mail.hust.edu.cn

Received
13th October 2011
, Accepted 22nd December 2011

First published on 31st January 2012

---

An economical route based on electrospinning and layer-by-layer (LBL) self-assembly processes has been developed to synthesize unique MoO2-modified TiO2 nanofibers, comprising a core of TiO2 nanofibers and a thin metal-like MoO2 nanolayer. The thickness of the MoO2 nanolayer can be tuned by altering the precursor concentration or the LBL cycles. When evaluated for their lithium-storage properties, the MoO2-modified TiO2 nanofibers exhibit a high discharge capacity of 514.5 mA h g−1 at 0.2 C over 50 cycles and excellent rate capability, demonstrating that enhanced physical and/or chemical properties can be achieved through proper surface modification.

---

## Introduction

The past decades have witnessed many significant advances in rechargeable lithium-ion batteries (LIBs) that are widely used in portable electronic devices and electric vehicles.1 In particular, recent progress in advanced nanoscience and nanotechnology is paving the way to the development of diverse nanostructured electrode materials for high-performance next-generation LIBs.2 Nanostructuring of electrode materials has led to improved energy density, higher rate capability, and better cyclability, due to the large surface area, numerous active sites, short mass and charge–diffusion distance, and efficient accommodation of volume changes.3 So far, a variety of TiO2-based nanostructures (e.g., nanoparticles,4 nanofibers5) have been fabricated as promising alternative anodes to carbonaceous materials in rechargeable LIBs because of their superior safety, low cost, chemical stability, and non-toxicity. Nevertheless, the main weakness of TiO2 lies in the intrinsically slow kinetics of Li+ diffusion (∼10−15 to 10−9 cm2 s−1) and low electronic conductivity (∼10−12 to 10−7 S cm−1), resulting in the deterioration of reversible capacity and rate capability.6 Therefore, considerable attempts (e.g., nanostructuring,7 doping,8 and surface coating9) have been made to overcome the ionic and electronic transport limitations of TiO2-based anodes.

Surface modification of electrode materials may not only promote faster Li+ diffusion or electron transport but also suppress the particle agglomeration and/or excessive growth.10 Also, it has been demonstrated that engineering the morphology of TiO2 into one-dimensional (1D) nanostructures (e.g., anatase nanorods11 and nanotubes,12 and TiO2(B) nanowires13) facilitates the electron transport along the long dimension and Li+ insertion/extraction along the lateral direction, and therefore enhanced electrochemical performances have been achieved. Inspired by this, we envision that the synergistic effects of nanostructuring and surface modification may contribute to the over kinetics of TiO2 as anode materials for lithium-storage applications.

Layer-by-layer (LBL) self-assembly is an attractive bottom-up approach to complex architectures and nanomaterials.14 Here we report on a facile LBL self-assembly approach combined with electrospinning for the fabrication of unprecedented MoO2-modified TiO2 nanohybrids. Onto the electrospun TiO2 nanofibers, positively charged polydiallyldimethylammonium chloride (PDDA) and negatively charged phosphomolybdate species were alternately adsorbed by the controlled LBL self-assembly process under ambient, aqueous conditions. After the post-heating treatment, MoO2-modified TiO2 nanofibers were achieved. Such a LBL processing is environmentally friendly, versatile, and low cost, and therefore offers potential for scalability to create desirable surface-modified electrode materials. To the best of our knowledge, this is the first report on the MoO2-modified TiO2 nanofibers through a LBL self-assembly process. In particular, metal-like MoO2 is an excellent host for lithium-ion storage, and has a theoretic capacity of 838 mA h g−1.15 Recently, we developed the electrospinning and self-assembly methods for the fabrication of carbon-coated MoO2 nanofibers,16 and hierarchical MoO2,17MoO2/graphene,18 and MoO2/C19nanostructures with enhanced lithium-storage performances. More interestingly, the present MoO2-modified TiO2 nanofibers, comprising a core of TiO2 nanofibers and a thin metal-like MoO2 nanolayer, exhibit enhanced electrochemical lithium-storage performances.

## Experimental details

### Preparation of TiO2 nanofibers

All chemicals were used as received without further purification. Deionized (DI) water was used throughout. Electrospun TiO2 nanofibers were fabricated as previously reported.20 In a typical procedure, a stock solution for electrospinning was prepared by dissolving poly(vinylpyrrolidone) (PVP, Mw ≈ 1300000, Aldrich), tetrabutyl titanate (TBT, 99.9%, Aldrich), absolute ethanol, and acetic acid under magnetic stirring overnight at room temperature. The concentration of PVP and TBT is 0.4 and 1.6 g mL−1, respectively. Then, the stock mixture was delivered by a computer-controlled syringe pump at a constant flow rate of 2 mL h−1, where the metallic needle was connected to a high-voltage power supply, and a piece of grounded aluminium foil under the needle was used to collect the samples. The fibrous TBT/PVP mat was formed at an electric field of 150 kV m−1 and dried at 80 °C for 24 h. Finally, TiO2 nanofibers were obtained by thermal treatment of the as-electrospun TBT/PVP mat at 500 °C (heating rate: 2 °C min−1) for 2 h in air.

### Preparation of MoO2-modified TiO2 nanofibers

The surface modification of the electrospun TiO2 nanofibers with metal-like MoO2 nanolayers was carried out by a LBL self-assembly technique combined with post-heating treatment. Scheme 1 illustrates the formation process for the MoO2-modified TiO2 nanofibers. Typically, 10 pairs of polyion (PDDA/PMA) layers were assembled on the surface of TiO2 nanofibers. PDDA (20 wt% in water, Aldrich) was used as the positively charged polyelectrolyte for alternate adsorption. The mat of the as-formed TiO2 nanofibers (0.1 g) was firstly immersed in a dilute PDDA solution (0.4 wt%) for 5 min. Subsequently, the TiO2/PDDA mat was soaked in an aqueous H3PMo12O40 (PMA, 10 mg mL−1) solution for 5 min. During each adsorption step, the mat was rinsed with DI water and dried. Then, the TiO2/(PDDA/PMA)10 composite was dried at 80 °C overnight, and annealed at 600 °C at a heating rate of 1 °C min−1 in H2(5%)/Ar for 5 h to produce MoO2-modified TiO2 nanofibers.
  

|  |  |  |
| --- | --- | --- |
|  | | |
|  | **Scheme 1**  Schematic representation for the preparation of the MoO2-modified TiO2 nanofibers by LBL self-assembly. |  |

### Materials characterization

Field-emission scan electron microscopy (FESEM) observations were carried out using a field-emission scan microscope (FEI, Sirion 200) coupled with an energy-dispersive X-ray (EDX) (Oxford Instrument) spectrometer. Transmission electron microscopy (TEM) and high-resolution transmission electron microscopy (HRTEM) were recorded by a JEOL JEM-2010F microscope. Powder X-ray diffraction (XRD) patterns were collected using an X'pert PRO (PANalytical B.V., Holland) diffractometer with high-intensity Cu Kα1 irradiation (λ = 1.5406 Å). The accelerating voltage and the applied current were 40 kV and 40 mA, respectively. X-Ray photoelectron spectroscopy (XPS) measurements were performed on a VG MultiLab 2000 system with a monochromatic Al Kα X-ray source (Thermo VG Scientific).

### Electrochemical measurements

The working electrodes were prepared by mixing 80 wt% active materials, 10 wt% acetylene black (Super-P), and 10 wt% polyvinylidene fluoride (PVDF) in N-methyl-2-pyrrolidinone. After coating the above slurries on Cu foils, the electrodes were dried at 80 °C in a vacuum for 6 h to remove the solvent. Then, the electrodes were cut into 8 mm disks and dried at 80 °C for 24 h in a vacuum. The coin cells were laboratory-assembled by a CR2032 press in an argon-filled glovebox where the moisture and oxygen were strictly limited to below 1 ppm. A lithium foil was used as the counter electrode, and a Celgard 2300 membrane was used as the separator. The commercial electrolyte was composed of 1 mol L−1LiPF6 in a mixture of ethylene carbonate (EC) and dimethyl carbonate (DMC) (v/v = 1:1). Cyclic voltammogram (CV) curves were recorded on a PARSTAT 2273 potentiostat at a scanning rate of 0.1 mV s−1 at room temperature. The galvanostatic discharge/charge tests were carried out on a multi-channel battery testing system (Land, China) with a cutoff voltage of 3.00–0.01 V vs. Li/Li+ at room temperature.

## Results and discussion

Fig. 1a and b show the FESEM images of the as-spun TBT/PVP nanofibers. Clearly, the surface of the product was smooth and these fibers were as long as several millimetres or even at the centimetre scale with the diameters of about 100–300 nm. Fig. 1c and d display the TiO2 nanofibers after thermally treating the electrospun TBT/PVP nanofibers at 500 °C for 2 h in air. It can be seen that the fibrous morphology was well maintained. Fig. 1e and f show the FESEM images of the MoO2-modified TiO2 product. By a LBL self-assembly process followed by the heating treatment, these TiO2 nanofibers were coated with a thin layer of MoO2. Evidently, the surface of the MoO2-modified TiO2 nanofibers becomes much rougher, compared with the TiO2 nanofibers. This indicates that a thin nanostructured layer of MoO2 has been successfully assembled on the surface of the TiO2 nanofibers. EDX analysis was also used to determine the local chemical composition of the TiO2 and MoO2-modified TiO2 products. As displayed in Fig. 2a for the TiO2 product, two major peaks correspond to the elements of Ti and O. Other peaks of C and Au were also observed, where the signal of C is generated from the conducting tape on the sample holder and the Au signal is generated from the Au particles used as a conductive coating for FESEM observations. In contrast, a clear peak of Mo appears in the MoO2-modified TiO2 product, which should come from the external layer of MoO2. The atomic percent of Mo is evaluated to be about 3.7%. In addition, the digital photos for the powdered samples of both the TiO2 nanofibers and the MoO2-modified TiO2 nanofibers were taken by a camera (Fig. S1, see ESI†). Furthermore, the thickness of the MoO2 coating can be tuned simply by controlling the concentrations of the PDDA and PMA solutions or the LBL cycles (Fig. S2, see ESI†).
  

|  |  |  |
| --- | --- | --- |
|  | | |
|  | **Fig. 1**   FESEM images of TBT/PVP nanofibers (a and b), TiO2 nanofibers (c and d), and MoO2-modified TiO2 nanofibers (e and f). |  |

  

|  |  |  |
| --- | --- | --- |
|  | | |
|  | **Fig. 2**   EDX spectra of the TiO2 nanofibers (a) and the MoO2-modified TiO2 nanofibers (b). |  |

The surface electronic states and chemical composition of the product were analyzed by XPS. As shown in Fig. 3a, the typical survey XPS spectrum of the MoO2-modified TiO2 nanofibers demonstrates the existence of Mo, Ti and O, which is consistent with the result of EDX. The high-resolution XPS spectrum of Mo 3d in Fig. 3b can be well fitted into two doublets. The peaks at 228.6 and 232.3 eV are for Mo(IV) 3d5/2 and Mo(IV) 3d3/2, which are characteristics of MoO2.21 Moreover, the doublet at 232.2 eV for Mo(VI) 3d5/2 and 235.6 eV for Mo(VI) 3d3/2 is detected, which may be resulted from the surface oxidation at the MoO2-modified TiO2 nanofibers in air.22 The high-resolution XPS spectrum of Ti exhibits two peaks at 464.3 and 458.7 eV with the separation of 5.6 eV. They are assigned to the Ti4+ oxidation state of TiO2 (Fig. S3, see ESI†).23

  

|  |  |  |
| --- | --- | --- |
|  | | |
|  | **Fig. 3**  (a) Survey XPS spectrum of the MoO2-modified TiO2 nanofibers. (b) High-resolution XPS spectrum of Mo 3d. |  |

The crystallinity of the TiO2 nanofibers and MoO2-modified TiO2 nanofibers was examined by XRD (Fig. 4). The diffraction peaks of the TiO2 nanofibers can be indexed to the anatase phase (JCPDS 21-1272) combining with the rutile phase (JCPDS 21-1276). The peaks at 25.28°, 37.80°, 48.05°, 62.69° and 70.31° are ascribed to the reflection of (101), (004), (200), (204), and (220) planes of anatase TiO2, and the peaks at 27.44°, 36.08°, 41.22°, 54.32°, 56.64°, 64.04° and 69.01° are ascribed to the reflection of (110), (101), (111), (211), (220), (310) and (301) planes of rutile TiO2. Compared with the TiO2 nanofibers, the peaks in the XRD pattern for the MoO2-modified TiO2 nanofibers become broader, indicating the nanocrystalline nature of the MoO2-modified TiO2 nanofibers. Owing to the annealing at 600 °C for 6 h, the part of the rutile phase in the MoO2-modified TiO2 nanofibers increases accompanying with decline of the anatase phase. The crystal phase of MoO2 is barely detectable in the XRD pattern of the MoO2-modified TiO2 product, arising from the small molar ratio of MoO2 (3.7%) under the detection limit of XRD.

  

|  |  |  |
| --- | --- | --- |
|  | | |
|  | **Fig. 4**   XRD patterns: (a) TiO2 nanofibers and (b) MoO2-modified TiO2 nanofibers. ★ anatase TiO2 and ◆ rutile TiO2. |  |

The microstructure of the TiO2 nanofibers and MoO2-modified TiO2 nanofibers was further investigated by TEM. It can be seen that the TiO2 nanofibers consist of nanoparticles with sizes ranging from 10 to 20 nm (Fig. 5a and b). In contrast to TiO2 nanofibers, the MoO2-modified TiO2 nanofibers exhibit a distinctively different mass contrast in the TEM images (Fig. 5c and d). A uniform MoO2 shell of 2–5 nm in thickness is coated on the surface of the TiO2 nanofibers, which agrees well with the FESEM results. Moreover, the SAED pattern of the MoO2-modified TiO2 nanofibers (Fig. 5e) reflects that the nanofibers are polycrystalline with the diffraction rings. Also, the HRTEM image of an individual MoO2-modified TiO2 nanofiber (Fig. 5f) displays three kinds of lattice fringes with lattice spacings of ∼3.5, 2.5 and 1.9 Å. They correspond to {101} planes of anatase TiO2, {101} planes of rutile TiO2, and {21} planes of MoO2, respectively.

  

|  |  |  |
| --- | --- | --- |
|  | | |
|  | **Fig. 5**   TEM images of (a and b) TiO2 nanofibers and (c and d) MoO2-modified TiO2 nanofibers. (e) SAED pattern and (f) HRTEM image of an individual MoO2-modified TiO2 nanofiber. |  |

Fig. 6a shows the first CV plot for the electrode of the MoO2-modified TiO2 nanofibers at a sweep rate of 0.1 mV s−1. The first discharge sweep exhibits three broad reduction peaks at 0.66, 0.97 and 1.65 V, where the peaks at 0.66 and 0.97 V reflect irreversible reduction of the electrolyte and the formation of a passivating surface film. The peaks at 1.65 V in the first discharge and 2.15 V in the first charge are associated with the Ti4+/Ti3+ redox couple during lithium insertion and extraction.11 From the second cycle onward, only the one couple at 1.65/2.15 V can be observed in Fig. 6b. The plots overlap well confirming the good reversibility of lithium extraction/insertion reactions.

  

|  |  |  |
| --- | --- | --- |
|  | | |
|  | **Fig. 6**  (a and b) CV curves of the MoO2-modified TiO2 nanofibers measured at a sweep rate of 0.1 mV s−1. (c) The discharge/charge profiles at a current rate of C/5 of the MoO2-modified TiO2 nanofibers. (d) Cycle life and rate capability of the MoO2-modified TiO2 nanofibers. |  |

The galvanostatic cycling of the electrode of the MoO2-modified TiO2 nanofibers was studied between 3.0 and 0.01 V at a current rate of C/5 (0.2 lithium per TiO2 in 1 h) (Fig. 6c). An evident potential plateau at about 1.75 V was observed in the first discharge curve, which is consistent with the previous results for TiO2.7a,11 Moreover, the first discharge capacity is as high as 680.5 mA h g−1, where the capacity between 1.0 and 0.01 V is 248 mA h g−1, about 36.6% of the total discharge capacity. After 5 cycles, the specific discharge capacity displays no decay but a slight increase, which has been described in other anode nanomaterials (e.g., MoO2 (ref. 15–18)). It may be attributed to a gradual activation process of the electrode made of the transition-metal oxide materials. The discharge capacity at the 50th cycle is 514.5 mA h g−1, and the capacity between the voltage range of 1.0 and 0.01 V is 308 mA h g−1, about 59.9% of the total discharge capacity. It indicates that more than half of the capacity for the electrode of the MoO2-modified TiO2 nanofibers comes from the voltage range of 1.0–0.01 V. This may result from the electrochemical properties of MoO2. Fig. 6d displays the rate capability and the cycle performance for the electrode of the MoO2-modified TiO2 nanofibers. The gradual increase of the specific capacity at the rates of 0.4, 1 and 2 C can also be observed. At the 50th cycle, the specific capacities at 0.4, 1 and 2 C are 372.7, 329.7, and 262.2 mA h g−1, respectively.

For comparison, the electrochemical properties of the TiO2 nanofibers were also explored. The theoretical capacity of TiO2 is 168 mA h g−1 (Li0.5TiO2).4–6Fig. 7a shows the first three CV curves of the electrodes made from the TiO2 nanofibers at a scan rate of 0.1 mV s−1. Compared to the MoO2-modified TiO2 nanofibers, the peak at 0.65 V in the first discharge of the TiO2 nanofibers is much sharper, which means that the irreversible reduction of the TiO2 nanofibers is more serious. Fig. 7b shows the discharge/charge curves for the TiO2 nanofibers at a current rate of 0.2 C over the potential range of 3–0.01 V. A potential plateau of ∼1.75 V appears clearly in the initial discharge cycle with a discharge capacity of 388.8 mA h g−1. However, the potential plateau at ∼2.1 V in the first charge curve shortens sharply with the charge capacity of 108.0 mA h g−1, indicating poor cyclability of the TiO2 nanofibers. From the second cycle onward, the capacity of the electrode decays continuously. The decline can also be proved from the CV investigations. From the second cycle onward, the discharge capacity decreases slowly and reaches 66 mA h g−1 at the 50th cycle. The electrochemical performance of the electrode of the TiO2 nanofibers is much worse when cycling at a high rate (Fig. 7c).

  

|  |  |  |
| --- | --- | --- |
|  | | |
|  | **Fig. 7**  (a) CV curves of the TiO2 nanofibers measured at a sweep rate of 0.1 mV s−1. (b) The discharge/charge profiles at a current rate of 0.2 C of the TiO2 nanofibers. (c) Cycle life and rate capability of the TiO2 nanofibers. |  |

The enhanced electrochemical properties of the MoO2-modified TiO2 nanofibers could be explained as follows: (i) with a MoO2 shell, the rough surface of the MoO2-modified TiO2 nanofibers could provide more active sites for holding Li+ ions than that of the TiO2 nanofibers with the relatively smoother surface, improving the capacity. (ii) MoO2, a metal-like electrical host for lithium storage, could not only improve the electrical conductivity, but also contribute to the specific capacity, especially for the voltage from 1 to 0.01 V. (iii) The TiO2 nanofibers can obtain a high discharge capacity of the first cycle. This is very important to weaken the irreversible reduction by the outer MoO2 shell and ensure the high reversible capacity from the second cycle onward.

Further work is underway to investigate the details of the synergistic effects of the nanostructured 1D TiO2 core and the nanoscale surface layer of MoO2 for lithium-ion storage applications. The electrochemical properties of the MoO2-modified TiO2 nanofibers that are prepared at the different concentrations of PDDA and PMA solutions are also examined. It is demonstrated that the amount of MoO2 is an important factor that dominates the electrochemical properties of the composite MoO2-modified TiO2 nanofibers (Fig. S4, see ESI†).

## Conclusions

In summary, unique MoO2-modified TiO2 nanofibers, comprising a core of TiO2 nanofibers and a thin metal-like MoO2 nanolayer, were synthesized via a facile and economical route based on an electrospinning process and layer-by-layer self-assembly for the first time. The thickness of the MoO2 nanolayer can be tuned simply by controlling the concentrations of the precursor solutions or the LBL cycles. Compared to the TiO2 nanofibers, the MoO2-modified TiO2 nanofibers show much better lithium-storage properties. The thickness of the MoO2 nanolayer affects the electrochemical properties of the MoO2-modified TiO2 nanofibers, demonstrating that enhanced physical and/or chemical properties can be obtained from proper surface modification. This route may also be extended to prepare other nanostructures with surface modification.

## Acknowledgements

This work was supported by the Natural Science Foundation of China (grant no. 51002057 and 50825203), the 863 program (grant no. 2009AA03Z225), the Natural Science Foundation of Hubei Province (grant no. 2008CDA026), and the PCSIRT (Program for Changjiang Scholars and Innovative Research Team in University). The authors thank Analytical and Testing Center of HUST for XRD, SEM and TEM measurements.


## Notes and references

1. (*a*) J. Y. Song, Y. Y. Wang and C. C. Wan, J. Power Sources, 1999, 77, 183 CrossRef CAS ;
   (*b*) J. M. Tarascon and M. Armand, Nature, 2001, 414, 359 CrossRef CAS ;
   (*c*) M. S. Whittingham, Chem. Rev., 2004, 104, 4271 CrossRef CAS .
2. (*a*) P. Poizot, S. Laruelle, S. Grugeon, L. Dupont and J. M. Tarascon, Nature, 2000, 407, 496 CrossRef CAS ;
   (*b*) E. Frackowiak and F. Beguin, Carbon, 2002, 40, 1775 CrossRef CAS ;
   (*c*) P. G. Bruce, B. Scrosati and J. M. Tarascon, Angew. Chem., Int. Ed., 2008, 47, 2930 CrossRef CAS .
3. (*a*) P. S. Herle, B. Ellis, N. Coombs and L. F. Nazar, Nat. Mater., 2004, 3, 147 CrossRef CAS ;
   (*b*) A. S. Arico, P. Bruce, B. Scrosati, J. M. Tarascon and W. Van Schalkwijk, Nat. Mater., 2005, 4, 366 CrossRef CAS ;
   (*c*) K. T. Nam, D. W. Kim, P. J. Yoo, C. Y. Chiang, N. Meethong, P. T. Hammond, Y. M. Chiang and A. M. Belcher, Science, 2006, 312, 885 CrossRef CAS .
4. Y. D. Wang, T. Chen and Q. Y. Mu, J. Mater. Chem., 2011, 21, 6006 RSC .
5. S. H. Nam, H. S. Shim, Y. S. Kim, M. A. Dar, J. G. Kim and W. B. Kim, ACS Appl. Mater. Interfaces, 2010, 2, 2046 CAS .
6. (*a*) M. V. Koudriachova, N. M. Harrison and S. W. de Leeuw, Phys. Rev. Lett., 2001, 86, 1275 CrossRef CAS ;
   (*b*) F. Gligor and S. De Leeuw, Solid State Ionics, 2006, 177, 2741 CrossRef CAS ;
   (*c*) S. Bach, J. Pereira-Ramos and P. Willman, Electrochim. Acta, 2010, 55, 4952 CrossRef CAS .
7. (*a*) J. S. Chen, Y. L. Tan, C. M. Li, Y. L. Cheah, D. Y. Luan, S. Madhavi, F. Y. C. Boey, L. A. Archer and X. W. Lou, J. Am. Chem. Soc., 2010, 132, 6124 CrossRef CAS ;
   (*b*) Y. S. Hu, L. Kienle, Y. G. Guo and J. Maier, Adv. Mater., 2006, 18, 1421 CrossRef CAS ;
   (*c*) D. V. Bavykin, J. M. Friedrich and F. C. Walsh, Adv. Mater., 2006, 18, 2807 CrossRef CAS .
8. (*a*) Y. D. Wang, B. M. Smarsly and I. Djerdj, Chem. Mater., 2010, 22, 6624 CrossRef CAS ;
   (*b*) Y. Furubayashi, T. Hitosugi, Y. Yamamoto, K. Inaba, G. Kinoda, Y. Hirose, T. Shimada and T. Hasegawa, Appl. Phys. Lett., 2005, 86, 252101 CrossRef .
9. L. J. Fu, H. Liu, H. P. Zhang, C. Li, T. Zhang, Y. P. Wu and H. Q. Wu, J. Power Sources, 2006, 159, 219 CrossRef CAS .
10. (*a*) S. Yoon, H. Kim and S. M. Oh, J. Power Sources, 2001, 94, 68 CrossRef CAS ;
    (*b*) A. Kannan, L. Rabenberg and A. Manthiram, Electrochem. Solid-State Lett., 2003, 6, A16 CrossRef CAS ;
    (*c*) K. Park, J. Son, H. Chung, S. Kim, C. Lee, K. Kang and H. Kim, Solid State Commun., 2004, 129, 311 CrossRef CAS .
11. X. Gao, H. Zhu, G. Pan, S. Ye, Y. Lan, F. Wu and D. Song, J. Phys. Chem. B, 2004, 108, 2868 CrossRef CAS .
12. J. Xu, C. Jia, B. Cao and W. Zhang, Electrochim. Acta, 2007, 52, 8044 CrossRef CAS .
13. (*a*) A. R. Armstrong, G. Armstrong, J. Canales, R. Garcia and P. G. Bruce, Adv. Mater., 2005, 17, 862 CrossRef CAS ;
    (*b*) G. Armstrong, A. R. Armstrong, P. G. Bruce, P. Reale and B. Scrosati, Adv. Mater., 2006, 18, 2597 CrossRef CAS ;
    (*c*) A. R. Armstrong, G. Armstrong, J. Canales and P. G. Bruce, Angew. Chem., Int. Ed., 2004, 43, 2286 CrossRef CAS ;
    (*d*) A. R. Armstrong, G. Armstrong, J. Canales and P. G. Bruce, J. Power Sources, 2005, 146, 501 CrossRef CAS .
14. (*a*) K. Ariga, J. P. Hill, M. V. Lee, A. Vinu, R. Charvet and S. Acharya, Sci. Technol. Adv. Mater., 2008, 9, 014109 CrossRef ;
    (*b*) Q. Ji, I. Honma, S. M. Paek, M. Akada, J. P. Hill, A. Vinu and K. Ariga, Angew. Chem., Int. Ed., 2010, 49, 9737 CrossRef CAS ;
    (*c*) M. Li, S. Ishihara, M. Akada, M. Liao, L. Sang, J. P. Hill, V. Krishnan, Y. Ma and K. Ariga, J. Am. Chem. Soc., 2011, 133, 7348 CrossRef CAS .
15. (*a*) Y. F. Shi, B. K. Guo, S. A. Corr, Q. H. Shi, Y. S. Hu, K. R. Heier, L. Q. Chen, R. Seshadri and G. D. Stucky, Nano Lett., 2009, 9, 4215 CrossRef CAS ;
    (*b*) Q. S. Gao, L. C. Yang, X. C. Lu, J. J. Mao, Y. H. Zhang, Y. P. Wu and Y. Tang, J. Mater. Chem., 2010, 20, 2807 RSC ;
    (*c*) Z. Y. Wang, J. S. Chen, T. Zhu, S. Madhavi and X. W. Lou, Chem. Commun., 2010, 46, 6906 RSC .
16. W. Luo, X. L. Hu, Y. M. Sun and Y. H. Huang, Phys. Chem. Chem. Phys., 2011, 13, 16735 RSC .
17. Y. M. Sun, X. L. Hu, J. C. Yu, Q. Li, W. Luo, L. X. Yuan, W. X. Zhang and Y. H. Huang, Energy Environ. Sci., 2011, 4, 2870 CAS .
18. Y. M. Sun, X. L. Hu, W. Luo and Y. H. Huang, ACS Nano, 2011, 5, 7100 CrossRef CAS .
19. Y. M. Sun, X. L. Hu, W. Luo and Y. H. Huang, J. Mater. Chem., 2012, 22, 425 RSC .
20. M. Shang, W. Z. Wang, L. Zhang, S. M. Sun, L. Wang and L. Zhou, J. Phys. Chem. C, 2009, 113, 14727 CAS .
21. J. F. Moulder, W. F. Stickle, P. E. Sobol and K. D. Bomben, Handbook of X-ray Photoelectron Spectroscopy, ed. J. Chastain, Perkin-Elmer Corporation, Eden Prairie, MN, 1992 Search PubMed .
22. S. H. Lee, Y. H. Kim, R. Deshpande, P. A. Parilla, E. Whitney, D. T. Gillaspie, K. M. Jones, A. Mahan, S. Zhang and A. C. Dillon, Adv. Mater., 2008, 20, 3627 CrossRef CAS .
23. G. Liu, W. Jaegermann, J. He, V. Sundstrom and L. Sun, J. Phys. Chem. B, 2002, 106, 5814 CrossRef CAS .

---

|  |
| --- |
| Footnote |
| † Electronic supplementary information (ESI) available: Digital photo, SEM, EDX, XPS, and electrochemical analyses. See DOI: 10.1039/c2jm15197a |

|  |
| --- |
| --- |
| **This journal is © The Royal Society of Chemistry 2012** |
